# Temping fates in Spain: hours and employment in a dual labor market during the Great Recession and COVID-19

**DOI:** 10.1007/s13209-021-00257-1

**Published:** 2021-12-02

**Authors:** Cristina Lafuente, Raül Santaeulàlia-Llopis, Ludo Visschers

**Affiliations:** 1 Universidad Carlos III de Madrid and European University Institute, Madrid, Spain; 2 Universitat Autonoma de Barcelona, BSE and CEPR, Cerdanyola del Vallès, Spain; 3 University of Edinburgh, Universidad Carlos III de Madrid, CESifo and IZA, Edinburgh, Scotland, UK

**Keywords:** Recessions, Dual labor markets, COVID-19, Spain, Furloughs, E32, J21, J64, J65

## Abstract

We investigate the behavior of aggregate hours supplied by workers in permanent (open-ended) contracts and temporary contracts, distinguishing changes in employment (extensive margin) and hours per worker (intensive margin). We focus on the differences between the Great Recession and the start of the COVID-19 Recession. In the Great Recession, the loss in aggregate hours is largely accounted for by employment losses (hours per worker did not adjust) and initially mainly by workers in temporary contracts. In contrast, in the early stages of the COVID-19 Recession, approximately sixty percent of the drop in aggregate hours is accounted for by permanent workers that do not only adjust hours per worker (beyond average) but also face employment losses—accounting for one-third of the total employment losses in the economy. We argue that our comparison across recessions allows for a more general discussion on the impact of adjustment frictions in the dual labor market and the effects policy, in particular the short-time work policy (ERTE) in Spain.

## Introduction

In Spain, employment responds strongly to downturns. This phenomenon is often linked to the dual nature of the Spanish labor market in which workers with temporary contracts (“temps”) coexist with workers with open-ended or permanent contracts (“perms”). The idea is that temps are much easier to lay off than perms and, hence, the flow out of employment during recessions is largely accounted for by temps (Bentolila and Dolado 1994; Bentolila et al. 2020).^1^ Hence, in a typical economic downturn such as the Great Recession, the loss of temp employment explains most of the loss in the aggregate hours of the total economy.^2^ We show that this is not the case in the COVID-19 Recession.

**Different response of the dual labor market to the Great Recession and COVID-19** In sharp contrast to previous recessions, we find that both perms and temps contribute to the loss of aggregate hours during COVID-19. Putting together the effects of employment and hours per worker, we show that perms account for the largest share—sixty percent—of the loss in aggregate hours in 2020Q2. This is explained by the fact that unlike in previous recessions: (1) Perms suffer losses in employment early in the recession—accounting for one-third of the total economy employment losses in 2020Q2, and (2) the COVID-19 response comes with a substantial downward adjustment in hours per worker that, at the same time, is larger for perms than for temps. These features persist and, one year into the recession, the larger relative contribution of perms to the loss of aggregate hours stands in 2021Q1.

In more detail, in the second quarter of the COVID-19 Recession—which largely overlaps with the national lockdown against the pandemic, we find that (weekly) aggregate hours in the total economy drop by four—an absolute deviation from their deseasonalized trend. This is a massive downward deviation from trend of approximately thirty percent on impact. The negative impact on aggregate hours is initially milder in the Great Recession, and it builds up slowly thereafter. In terms of the dual labor market behavior, we find substantial differences between the aggregate hours of perms and temps across these two recessions. While only the aggregate hours of temps react—and gradually so—during the first year of the Great Recession, the aggregate hours of both perms and temps drop on impact in the COVID-19 Recession. Further, perms account for the largest share of the drop experienced in the total economy upon impact and this is still the case by 2021Q1.

The explicit distinction between employment and hours per worker separately for perms and temps turns out to be critical in order to understand the differential drivers behind the behavior of aggregate hours across recessions. In 2020Q2, employment (per working-age population) drops by 4.3 percentage points in the total economy and perms account for approximately one-third of that loss. In terms of hours per worker, we find an unprecedented large drop of 6.5 (weekly) hours per worker in the total economy. Splitting the sample between perms and temps, we find that perms drop 7.1 hours per worker—larger than the average adjustment in the total economy—whereas temps adjust their hours per worker downward by 5.5. These patterns are in stark contrast to the Great Recession which was largely accounted for by employment losses—hours per worker did not adjust—and initially mainly by temps.

**Why the different response: the nature of the recession or policy?** A potential rationale for the different responses of the dual labor market across recessions is the different nature of the Great Recession and the COVID-19 Recession. This differential nature shows in the heterogeneous response of the labor market across industries. First, in the onset of the Great Recession the construction sector was clearly the most affected. This sector also had large shares of temp workers, which explains the sharp instant decline of temp hours and employment, while perms were relatively unaffected in the first quarters of the recession. Manufacturing industries related to construction also suffer more, and after some quarters, the financial sector was also largely affected. But for the rest of the economy, it took time to build up the losses. In contrast, in the COVID-19 Recession hospitality and retail were by far the most affected—both because of the fall in demand for in-person services and because of the forced closures during lockdown.^3^ In contrast, industries that could shift to remote work and those deemed essential activities were less affected by the COVID-19 crisis, in particular, earlier in the recession. At the same time, the COVID-19 Recession differs from previous recessions not only in its nature—asymmetrically affecting industries in manner that differs by recession—but also in the set of unprecedented economic policies put forth to prevent business closures and employment losses. In particular, a new short-time work policy (Expediente de Regulación Temporal de Empleo, ERTE) was instated few days after the beginning of the national lockdown.^4^

Here, we show that the aggregate patterns that we document on the behavior of employment and hours of perms and temps across recessions stand within industries. We take this finding as suggestive evidence that the differential response of the dual labor market across recessions is likely due to policy rather than the different nature of the recessions. This includes the set of policies implemented to cushion the COVID-19 Recession, in particular, the ERTEs. However, identifying the isolated effects of ERTEs is problematic given the package of alternative policy measures that was implemented almost simultaneously; see our discussion in Sect. 2.3.^5^ In this context, in order to explore the potential role of the ERTEs we simply re-conduct our analysis removing from our sample of employed individuals the population that reports being under ERTEs. This exercise helps us highlight the different behavior of employment (and hours per worker) of the actual economy (benchmark sample) with ERTEs versus an alternative view of the same economy where the individuals under ERTE are not considered employed (i.e., a restricted sample that excludes individuals with ERTEs from the employed population).

With this exercise, we find that the ERTEs, in addition to preventing employment losses, help explain approximately half of the drop in hours per worker. This reasoning is also in Eyméoud et al. (2021a) drawing from an international comparison between the USA and some countries in the European Union that implement short-time work policies. At the same time, our analysis shows that the ERTEs do not sustain a scenario whereby the sole margin of adjustment is in hours per worker. In particular, perm workers suffer large employment losses even with ERTEs during COVID-19. However, the duality of the labor market is still present during COVID-19 in the same direction as in the Great Recession in the sense that temps show larger employment losses than perms in relative terms, as percentage deviations from their respective trends, by a factor of five. Interestingly, we find that this duality—or inequality—result in terms of employment is in large part sustained by the ERTEs that asymmetrically benefit perms. Indeed, in the restricted sample without ERTEs, the differential factor of employment losses between perms and temps drops from five to two. Further, the more flexible adjustment of hours per worker cannot be fully attributed to the ERTEs since hours per worker also substantially drop for individuals without ERTEs. With or without the ERTEs, we find similar magnitudes in the drop of hours across perms and temps. Part of the drop in hours per worker that we document for the sample economy without ERTEs could be partly explained by the 2012 reform that not only lowered the lay-off costs for perms but also increased the ability to re-negotiate at the firm level the collective sector-level agreements regarding hours which increased the flexibility of hours per worker (Doménech et al. 2018).

**Insights from the aggregate hours of perms and temps and a static model** Acknowledging the limited ability to empirically assess the impact of the ERTEs in explaining the different behavior of the dual labor market across recessions, we further assess the hypothesized mechanism for ERTEs constructing a static model—with some degree of complementarity between the perms and temps. Since the model is based on aggregate hours separately for perms and temps, we provide additional empirical insights that describe the complete path of aggregate hours by type of worker during the Great Recession and the COVID-19. Empirically, we show that the negative impact on perms took years to build during the Great Recession, but occurred right at the start of the recession during COVID-19. This implies that early in the Great Recession—approximately the first six quarters that follow 2008Q1, the ratio of temp hours to perm hours declines noticeably, whereas the level of perm hours barely changes. That is, the adjustment comes from temp hours early in the Great Recession. In a second part of the recession, from approximately 2010Q2 to 2013Q2, the decline in the temp to perm hours ratio slows down. Since permanent hours drop more noticeably in this second part of the recession, the margin of adjustment flips from temps to perms. As a result, we find a *J*-pattern between the temp-to-perm hours ratio and the hours of permanent workers during the Great Recession in Spain from 2008 to 2013. Then, in the initial phases of the recovery—from 2014 to 2016—the ratio of temporary to permanent hours grows at the same time that perm hours increase. Hence, the evolution of temp hours and perm hours during the Great Recession and its recovery are well summarized by a *clockwise loop* pattern. As we document, these features are largely shared across industries.

We find that our model can generate the clockwise hours adjustment that we document for the dual labor market during the Great Recession (a high labor adjustment friction scenario). Then, we use this framework as a device to explore how the ERTEs (that ease the labor friction) can help explaining the patterns of aggregate hours of perms and temps during COVID-19. By construction, this model abstracts from any dynamic considerations such as the fact that firms potentially face a dynamic trade-off between having a stable workforce—saving on hiring costs and accumulating skills—and their ability to adapt to negative shocks. In our static setting, this trade-off is partly captured—in a reduced form manner—through a technological complementarity between temps and perms.^6^ Qualitatively, we find that the introduction of the ERTEs in the model delivers similar dynamics as those that we document for the COVID-19 Recession which, we believe, reassures the role of the ERTEs in helping explain the documented patterns. We leave a more careful quantitative analysis of the ERTEs with dynamic considerations for future research.

**Further empirical insights from business dynamics and parents labor supply** Although we cannot empirically identify the causal channels behind of the large differences between the dual labor market behavior during the Great Recession and COVID-19, the distinct set of policies instituted during COVID-19 intuitively affect the labor market in ways that line up with the empirical patterns we document. First, despite the initial response with a national lockdown of non-essential business activity, we do not see larger employment losses—neither business closures—during COVID-19 than in the Great Recession. A natural candidate for this result is the ERTEs. The ERTEs can lower the operational costs of businesses—allowing firms to stay afloat—with a contractual arrangement that aims to reduce layoffs for a period of, initially, 6 months.^7^ Further, in contrast to the Great Recession, we also find a reduction in business closures during COVID-19 which are also likely driven by a combination of the ERTEs and additional financial aid measures—credit—to firms. Second, another channel that may explain the sharp employment and hours losses in 2020Q2 is the effect of school closures on labor supply.^8^ Our data do not allow us to identify households with school-aged children, so instead we proxy that variable by focusing on the middle-aged married workers. Married individuals suffer larger hours and employment losses than singles, with married women in particular being most affected. However, these differences are relatively small in the aggregate and thus we conclude that school closures and other care duties during lockdown are unlikely to explain the fall in hours we observe—although they may help explain the impact of the recession on parents, particularly women.

We discuss our data and the institutional background in Sect. 2. In Sect. 3, we describe the dual labor market behavior from the late 1980s to the present, compare its response to the Great Recession and COVID-19, and provide a an exploratory discussion on the role ERTEs in explaining the observed patterns. In Sect. 4, we briefly outline a simple static model that helps us rationalize some of the patterns that we uncover. In Sect. 5, we conduct a robustness cross-industry analysis and discuss further context in terms of the role of policy on business closures (and formation) and paternal labor supply. Our last section concludes.

## Data and context

In this section, we first describe the data we use in our analysis in Sect. 2.1. Second, we discuss the institutional context of labor market duality in Sect. 2.2 and summarize the COVID time line in Spain in Sect.  2.3.

### Data

We use the confidential, expanded version of the Spanish labor force survey (Encuesta de poblacion activa, EPA) provided by the National Statistics Institute (INE). This version differs from the freely available version in that it contains more detailed data, including anonymized household identifiers that allow us to connect households interviewed in consecutive surveys. The survey has a quarterly rotating panel structure with over 100,000 observations per quarter, where households are followed for six quarters.^9^ Representativity is kept through population weights, which we will apply throughout this paper. Our data start in the second quarter of 1987 and go until the first quarter of 2021. These data are used by Eurostat and other international organizations such as the OECD or the World Bank.

Like other datasets of its kind, the EPA has suffered changes in survey design though the years. The most important ones happened in 1992, 1999, and 2005. For the purpose of this paper, the most relevant change is the 2005 redesign, which modernized the interview process, increased its coverage, and adapted the sample to make it more representative of the population in 2005—most notably, to take into account the substantial demographic changes in Spain in the early 2000s. The changes in the survey improved the coverage of short employment spells and marginally attached workers. The change was implemented in a way which did not affect the stocks of employed workers, but it did create a noticeable discontinuity in the labor state flows [see Lafuente (2020) for more details]. In this context, it is particularly interesting for our study the fact that we do not find discontinuities in the series of employment or hours that emerge from the EPA. We do find two minor changes: a change in temporary employment and a minor break in hours per worker (as reflected in Fig. 2). The discontinuity in hours per worker is driven by the increased weight given to respondents engaged in casual work. Since we mostly focus on the 2008 and 2020 recessions, our study is not affected by these survey changes insofar we use data starting in 2005Q1 in order to generate the projected counterfactual trends of the 2008 recession. In the instances where we use a longer time series before 2005Q1, we correct for the survey changes with a dummy in 2005Q1—net of a trend within a two-year window from 2004Q1 to 2006Q1—which captures the discrete change observed in that quarter.

**Labor market variables** We focus on aggregate hours per working-age population (*H*) defined as the product of employment per working-age population (*e*) and hours per worker (*h*).^10^ Then, taking into account the dual market with two types of workers, perms (*P*) and temps (*T*), the aggregate hours in the total economy are,

$$\begin{aligned} H = H_P + H_T = h_Pe_P + h_Te_T. \end{aligned}$$

Further, note that we can rewrite $H = he$ where average hours per worker in the aggregate economy are weighted by the share of each type of employment $h = h_Ps_{e,P} + h_Ts_{e,T}$ with $s_g=\frac{e_g}{e}$ and $g=\{P,T\}$, that is,

$$\begin{aligned} H = he = (h_Ps_{e,P} + h_Ts_{e,T})e, \end{aligned}$$

which implies that we can describe the behavior of aggregate hours by understanding the behavior of *e*, the employment shares of *P* and *T*, and the hours per worker of *P* and *T*.

### Labor market duality

Since the de-regularization of temporary contracts that took place in 1984, Spain has become a stand-out case of dual labor markets in the OECD. With unemployment soaring over 20% at the time, Spain sought to introduce *flexibility at the margin*: preserving very stringent employment protection (EPL) for regular workers, while allowing firms to hire through temporary contracts with very little dismissal costs (Bentolila and Dolado 1994). Before the 1984 reform, only certain industries and under special circumstances were allowed to use these contracts. In the years after the reform, the contracts became widely used: By 1992, more than 30% of all workers were in temporary contracts, as the bottom right panel of Fig. 2 shows.

With the drawbacks of temporary contracts becoming more apparent, attempts to substantially lower the reliance on temporary contracts have proven less successful, though some reduction has been achieved over the next 30 years. In the time series in Fig. 2, we can observe the relatively modest success of the reforms (Dolado et al. 2002) in the 1990s. Then, in the first half of the 2000s the construction sector, in which temporary work was most prevalent, grew considerably faster than others, pushing up the temporary-to-permanent ratio [see Dolado et al. (2002) for more details]. A further reform in 2006 generated an impact, e.g., through ending the social security subsidies for conversions of temporary to permanent employment by the end of the year 2007, generating a substantial spike in conversions to permanent (visible in the bottom and upper left panels of Fig. 2 between 2006 and 2007), but with short-lived effect.^11^

After the Great Recession of 2008, a new labor reform was announced in 2012. Crucially, this reform allowed greater powers to firms to set the working conditions, which was reflected in wages but also in hours: There is evidence that the reform increased the use of part-time work and other flexible hours arrangements [see, e.g., Doménech et al. (2018) and Stepanyan and Salas (2020)]. However, even though it introduced a reduction in the severance payments for permanent workers, the reform failed to solve the duality of the labor market, [see Bentolila et al. (2012b), Bentolila et al. (2012c) and Bentolila et al. (2020)]. These cuts left firing costs that still appear to be sufficiently high to push firms toward using temporary contracts as their main margin of adjustment (Bentolila et al. 2020). Overall, over the 2010s, the share of temporary workers and hours has grown back slightly to about 25% of the total, so duality remains an important feature of the Spanish labor market, and even more so for the young and less educated workers.

Temporary contracts with strict limitations on their renewal, as in Spain, have been associated with many labor market distortions (e.g., in terms of human capital accumulation, the technology and skill mix, and intergenerational inequality and equity (Alonso-Borrego et al. 2005; Caggese and Cuñat 2008; Cahuc et al. 2016; Caggese et al. 2019; Guner et al. 2020). These considerations do not only operate in the long run, but also in response to short run shocks. Below, we compare and contrast the empirical patterns of adjustment of temporary and permanent work during the crisis years (2008–2013) with the COVID Recession, also to help inform theories and measurement of these distortions.

### The COVID-19 crisis in Spain

As the COVID-19 impact came with various time-varying restrictions on production, labor supply, and demand, we first summarize the COVID-19 time line in Spain before spelling out in more detail the ERTE policy.

#### A time line of the epidemic

The first confirmed COVID-19 infection in Spain happened on January 31, 2020.^12^ During the month of February, cases were mostly imported, which prompted the chief of the Coordination Centre for Sanitary Emergencies (Centro de CoordinaciAn de Alertas y Emergencias Sanitarias), Fernando SimAn, to reassure the population that Spain would see mostly a few imported cases, but it would not become significantly affected by the pandemic. However, the first known locally transmitted case was recorded on 26 February. Confirmed cases increased exponentially subsequently: They grew from 73 on 1 March to 589 on 8 March to 5,753 on 14 March. This leads to the government declaring the state of alarm on 15 March and implements a national lockdown. This came almost a week after Italy declared a similar lockdown on 9 March.

The lockdown was supposed to last 15 days, but it was effectively extended until 21 June, thus covering nearly all of the second quarter. Some restrictions were eased progressively. From 15 March until 13 April, all non-essential economic activities were closed down. After that day, workers in some non-essential sectors, such as construction and industry, and those who could not work remotely were allowed to return to work. Schools remained closed, however.

After a less restricted summer, the start of the second wave in the fall precipitated the return to the state of alarm on 24 October. It was only lifted on May 9, 2021. However, this lockdown measures were softer, with most economic activities except the hospitality sector allowed to carry out as normal. The management of the restrictions was also decentralized to the regional level, resulting in substantial heterogeneity in restrictions in the second lockdown.

**Fig. 1:**
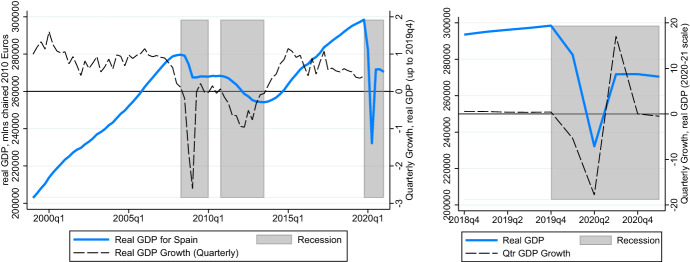
GDP of Spain across recessions (right panel: COVID Recession outtake). Note: GDP Figures from Eurostat (2021). The units are millions of chained 2010 Euros, seasonally adjusted

In Fig. 1, we plot the evolution of the real gross domestic product in Spain over the (technically) twin recessions that we consider jointly as the “Great Recession” (from the second quarter of 2008 to the second quarter of 2013), and the COVID Recession (which followed the peak in the fourth quarter of 2019).^13^ Since the latter involves large swings from quarter to quarter, which dwarf the smaller but more persistently accumulating negative quarterly growth rates during the Great Recession, we present a separate graph of our last ten quarters (2018Q4–2021Q1) under consideration in the right panel. During COVID times, we observe a large drop, nearly 18%, in the second quarter, followed by a large, but still incomplete recovery in 2020Q3. This, in turn, is followed by a small subsequent drop in GDP, during what was the second state of alarm.

#### The ERTEs

In Spain, an important response to the economic impact of the COVID crisis was regulated by the Real Decreto-ley 8/2020 on 18 March—4 days after the imposition of the national lockdown. Most notably, this law introduced the ERTEs, a short-time work policy that allows firms and workers to agree to a suspension of employment where the worker still receives a proportional amount of her regular salary (typically 70%), paid from her social security contributions. In regular times, the worker would be consuming her own unemployment insurance allowance for the duration of an ERTE. Instead, during the COVID Recession, the worker can use the ERTE preserving her unemployment insurance time allowance, while the firm saves the social contributions it pays—as long as it does not dismiss any worker in the 6 months after the end of the short-time work period.^14^$$^,$$^15^ For temporary workers, their contracts are automatically extended for the duration of the short-time work scheme. If the firm fires *any* worker (before their contract ends) in the 6 months after the first worker is back to work from short-time work, all subsidies perceived by the firm must be returned in full to the administration. However, it is worth noting that the firm can chose to not renew any temporary contracts that expire in those 6 months.^16^ For the purposes of this paper, we treat ERTE as creating flexibility on the hours margin, without dismissal, for the quarters under consideration.

Other important economic measures included 100.000 million euros for firms and self-employed workers to use as co-lateral for credit, and 10.000 million euros extended to ICO, the public credit institute, for immediate liquidity needs of small and medium enterprises (SMEs). The deadline for the payment of taxes and duties was extended as well.^17^

#### Timing of economic policy and the lockdown

The frequency of the data for this paper is quarterly,^18^ which implies that we cannot separate the impact of the COVID crisis (which started in late February), the impact of the lockdown (declared in mid-March, phased out May–June) and the impact of the economic measures to support workers affected by the lockdown (which happened a few days after the lockdown was declared). It all happened between the end of the first quarter of the year 2020 and the second. While the impact in the second quarter is very clear-cut, the first quarter was a period of uncertainty driven by the expectation of the government that the pandemic was not going to severely hit the population and the economy.^19^

Overall, the economic impact of COVID in Spain has been threefold. First, the direct impact is due to sick leave and mortality of infected people. Second, the economic effects are due to restrictions on economic activities. As mentioned above, prominent among these the brake on all non-essential economic activity^20^ during the lockdown, mostly in the second quarter of 2020; and later, the much more partial brake on economic activity during the second state of alarm from the fourth quarter of 2020 onward. Restrictions on international tourism, also in origin countries of tourists, and more generally behavioral responses of economic agents to COVID, have had a further significant impact beyond the states of alarm themselves. Third, economic policy measures have been instituted to counteract the adverse impacts of COVID and associated restrictions, among these, importantly, the extraordinary flexibility measures such as the ERTEs (which are ongoing beyond the first quarter of 2021) and the banning of economic dismissals in the second quarter of 2020.

## Trends and cycles of the dual labor market

First, we study the trend behavior of aggregate hours, employment, and hours per worker separately for perms and temps since the late 1980s to the most recent available data in 2021 (Sect. 3.1). Second, we look into the cyclical behavior of the Spanish labor market focusing on the two most recent recessions including the financial crisis in 2008 and the COVID-19 crisis and provide a discussion on the role of the ERTEs in this context (Sect. 3.2). Third, we conduct a cross-industry analysis over time (Sect. 6.1).

**Fig. 2:**
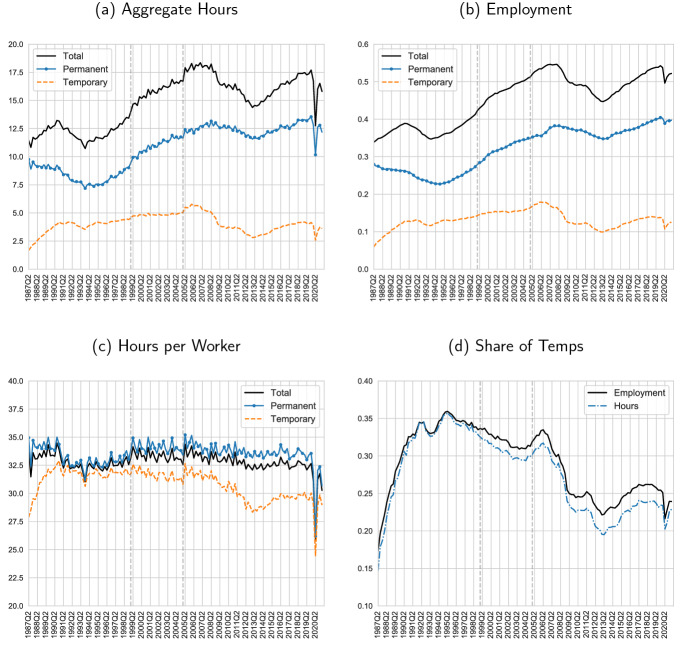
Hours and employment: total, perms, and temps, Spain 1987Q2–2021Q1. *Notes:* Aggregate hours and employment are divided by working age population. All variables are reported on a weekly basis. The vertical dashed lines correspond to the quarters in which the EPA survey was re-designed as described in the text

### Three-decade trends

We show the time series of aggregate (weekly) hours and its components for the total economy as well as separately for perms and temps in Spain 1987Q2 to 2021Q1 in Fig. 2. Focusing on the trend behavior of aggregate hours per working-age individual, we find a substantial increase over the past three decades. Precisely, aggregate hours per working-age adult are 11.7 in 1987Q2 increasing to 17.6 in 2019Q1; see panel (a) in Fig. 2. Aggregate hours are normalized by working-age population (16+) and reported per week. We keep this normalization throughout the entire paper.

The largest part of these three-decade increase in the total economy aggregate hours—approximately two-thirds—is accounted for by the perms. In 1987, aggregate permanent hours, i.e., the sum of all hours by permanent workers normalized by working-age population, were 9.9 hours rising to 13.0 in 2019. The aggregate temporary hours have increased from 2.3 in 1987 to 3.8 in 2019, though in a clear non-monotonic fashion. To be clear, both total permanent and temporary hours are normalized by the same denominator (total working-age population) and hence are additive component of overall aggregate hours in the previous paragraph.^21^

The peak for the entire time series of aggregate hours is reached right before the Great Recession: 18.0 for the total economy-wide hours (2007Q1), 12.7 for perm hours (2008Q2), and 6.0 for temp hours (2007Q1). Further, note that aggregate hours in 2019, before the COVID-19 Recession—have not yet recovered their pre-Great Recession levels in the total economy, with a sizeable gap (relative to the pre-Great Recession peak) in total temp hours persisting more than a decade later.

The trend behavior of aggregate hours is largely explained by employment; see panel (b) in Fig. 2. We again normalize employment by the working-age population. Employment, as a proportion of the working-age population, has dramatically increased from 34 percent in 1987 to 56 percent in 2019—with a peak close to that figure before the Great Recession. The patterns of employment for perms and temps largely mirror the patterns of their respective aggregate hours. In particular, the three-decade increase in employment in the total economy largely originates in the perms.

In contrast to the behavior of aggregate hours and employment, hours per employed worker (often abbreviated as “hours per worker”) are remarkably constant over the past three decades fluctuating around 33; see panel (c) in Fig. 2. Perms show similar weekly hours per worker to those of the total economy throughout, slightly larger from the early 2000s. On the contrary, temps show a more pronounced decline from an average of 33 weekly hours per worker in the early 1990s to 29 weekly hours per worker in the late 2010s. The decrease in the temps’ weekly hours per worker occurs from the early 2000s to the mid-2010s after which the temps’ weekly hours per worker remain relatively steady around 29 weekly hours until the COVID-19 Recession.

In terms of the share of aggregate hours by temps and perms, total temp hours account for an average of 28 percent of aggregate hours over the entire period, growing from less than 20 percent in the late 1980s to levels above 30 percent through the 1990s, after which temps account for approximately 25 percent of aggregate hours since the aftermath of the Great Recession; see panel (d) in Fig. 2. The share of employment by temps is similar to that of aggregate hours except for a slight larger drop in the share of aggregate hours since the early 2000s.

Aggregate hours in Fig. 2 show large fluctuations since the late 1980s. In particular, although the years prior to the Great Recession and the most recent COVID-19 Recession aggregate hours increase in relatively steady manner, aggregate hours substantially decline during both recessions, but with very different underlying dynamics, which we address in more detail next.

### The Great Recession and COVID-19

Here, we assess the effects of a recession on aggregate hours and its components separately for perms and temps. For this end, we compute the absolute deviations between the actual value of a variable of interest during a recession and an empirical “counterfactual” that captures the value that the variable of interest would attain had the recession not occurred. To construct this “counterfactual,” we project the predicted values of a pre-recession deseasonalized trend (of a variable of interest) onto the recession quarters.

**Fig. 3:**
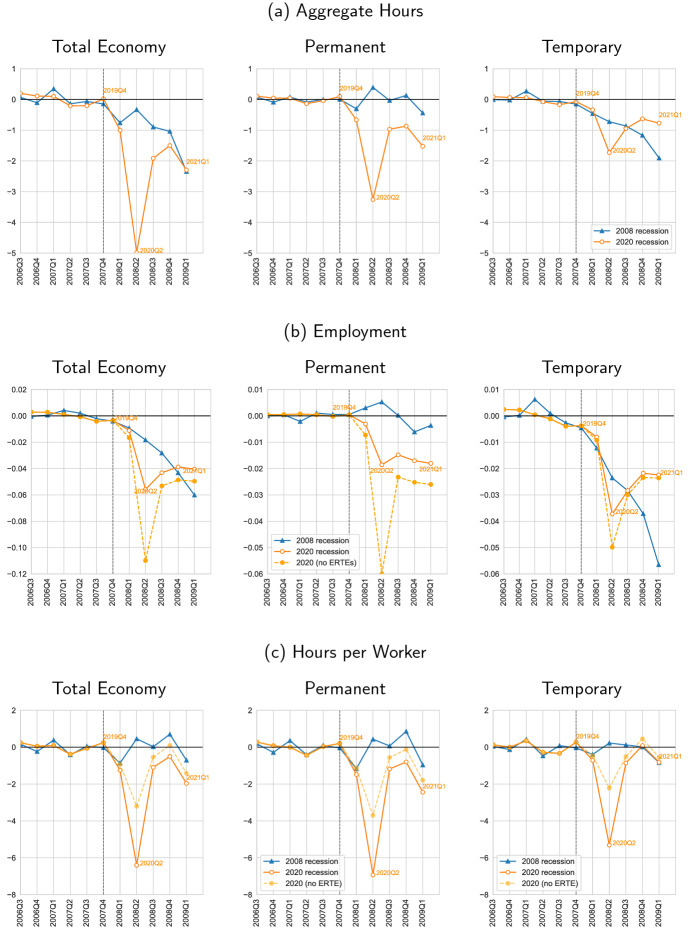
Response of the dual labor market, *absolute* deviations: Great Recession and COVID-19. *Notes:* All variables are plotted as level deviations from a predicted trend from 1 that uses data up to the onset of recessions. Aggregate hours (*H*) and employment (*e*) are divided by the working age population. Aggregate hours and hours per worker (*h*) are expressed in a weekly basis. Note that we have shifted the Covid-19 Recession in time in order to normalize the onset of the Covid-19 Recession (solid orange line) to that of the Great Recession (solid blue line). The vertical dashed line denotes the normalized onset of both recessions. We also add a alternative "No ERTE" path where we drop individuals with ERTEs from the employment series (dashed yellow line) (color figure online)

That is, for our purposes, the difference between the actual data during the recession and the projection of a pre-recession (deseasonalized) trend onto the recession quarters captures the effects of the recession, as, e.g., in Eyméoud et al. (2021b). Note that the empirical “counterfactual” (i.e., the predicted value) is a projection from an estimation that uses strictly only pre-recession data. Precisely, for quarters $t<t_R$, where $t_R$ is the quarter where a recession starts, we estimate the pre-recession deseasonalized trend as:

1 $$\begin{aligned} x_{t} = cons. + \gamma \; t + \delta _Q {\mathbf {1}}_t + e_t, \end{aligned}$$

with $x_{t} = \{H_{t},e_{t},h_{t}\}$. There is an time trend captured by $\gamma$ and a seasonal component captured by the quarterly dummy coefficients $\delta _Q$. Then, we construct the predicted values ${\widehat{x}}_t = \widehat{cons.} + {\widehat{\gamma }} \; t + {\widehat{\delta }}_Q {\mathbf {1}}_t$ for all periods including their projection for $t \ge t_R$ onto the recession quarters. The difference between the predicted value ${\widehat{x}}_t$ (including its projection onto the recession quarters) and the actual value $x_t$, i.e., $\Delta _t = {\widehat{x}}_t - x_t$, is what we plot in Fig. 3. Note that we conduct this exercise separately by recession. Specifically, we use an onset of the recession, $t_R$, equal to 2008Q1 for the Great Recession and equal to 2020Q1 for the COVID-19 Recession. In each recession, we use the several quarters before $t_R$ to estimate (1) spanning from 2005Q1 to 2007Q4 for the Great Recession and from 2013Q1 to 2019Q4 for the COVID-19 Recession.

Our results are in Fig. 3 separately for aggregate hours and its components: employment and hours per worker. The analysis is decomposed across perms and temps. Further, to ease the comparison between the response across the two recessions we shift the time of the onset of the COVID-19 Recession (see dashed vertical line) in order to make it coincide with the onset of the Great Recession; see Fig. 3. In this manner, our strategy to compare the dynamic (IRF-like) responses of the labor market across recessions resembles an event study—indeed, before the start of each recession, we show that the deviations from trend of the labor market variables are similar across recessions. Unfortunately, as we discussed earlier, the combination of overlapping policies that are put forward in response to COVID-19 makes it challenging to attribute the differential response across recessions to a specific policy. Nevertheless, as a first attempt to assessing the role of ERTEs we also reconduct our analysis by dropping the individuals with ERTEs from our sample of employed individuals. This analysis helps us gain insights on the role of ERTEs in preventing the loss of employment and in generating the drop of hours per worker during the COVID-19 Recession.

**Aggregate hours** We find that during the COVID-19, there is a loss of 4 aggregate hours (per working-age individual) in the second quarter of the recession, 2020Q2; see left column in panel (a) in Fig. 3. This implies a massive deviation of aggregate hours of approximately 30 percent below trend in 2020Q2. In contrast, the effects that we observe in the Great Recession are slower and initially milder than in the COVID-19 Recession. In particular, three quarters into the Great Recession we find a loss of 2 aggregate hours—i.e., half of the total drop observed during the second quarter into the COVID-19 Recession. With the end of the lockdown, the Spanish economy starts to partially in 2020Q3 reducing the loss of aggregate hours below trend, and in 2020Q1, the aggregate hours loss coincides with that of the Great Recession in 2009Q1, a response that builds up slower.

Interestingly, by splitting the sample between perms and temps we find that these components of the behavior of aggregate hours substantially differ across recessions. During the initial COVID-19 hit, approximately 60 percent of the drop in aggregate hours is driven by perms, who experience a drop of 2.5 aggregate hours (again in “per working-age individual” units); see the center column in panel (a) in Fig. 3. The remaining 40 percent is due to temps, whose aggregate hours drop by 1.5; see the right column in panel (a) in Fig. 3. During the subsequent quarters, working hours for temporary and permanent recover only partially, i.e., are still substantially below the pre-COVID trend.

In this manner, the labor market response of the COVID-19 Recession shows a behavior that contrasts strongly with previous downturns. For example, during the Great Recession total *permanent* hours were not affected one year into the recession; there, the drop in total temporary hours is almost entirely responsible for the decline in the whole economy. Differently, the response of aggregate hours is more symmetric in the COVID-19 Recession than what it was in the Great Recession; if anything, the COVID-19 Recession shows a larger loss for perms than for temps one year into the recession, in sharp contrast to previous downturns.

**Fig. 4:**
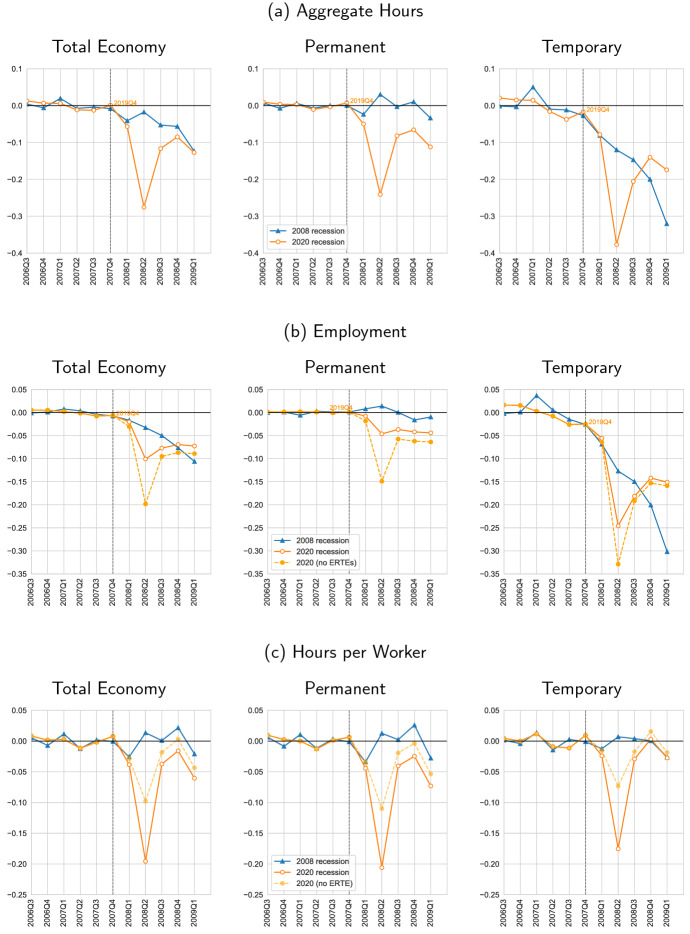
Response of the dual labor market, *relative* (%) deviations: Great Recession and COVID-19. *Notes:* All variables are plotted as *relative* (%) deviations from a predicted trend from 1 that uses data up to the onset of recessions. Aggregate hours (*H*) and employment (*e*) are divided by the working age population. Aggregate hours and hours per worker (*h*) are expressed in a weekly basis. Note that we have shifted the Covid-19 Recession in time in order to normalize the onset of the Covid-19 Recession (solid orange line) to that of the Great Recession (solid blue line). The vertical dashed line denotes the normalized onset of both recessions. We also add a alternative "No ERTE" path where we drop individuals with ERTEs from the employment series (dashed yellow line) (color figure online)

**Employment** The employment losses during COVID-19 and the Great Recession are in panel (b) in Fig. 3. During the second quarter of the COVID-19 Recession, we find a drop of approximately 6 percentage points of employment (relative to the working-age population). This implies a drop of approximately 10% in percentage deviation from trend employment; panel (b) in Fig. 4. The Great Recession shows a ballpark similar drop in terms of employment in the first year, accumulated at a slower pace, but persistently so; see the left column of panel (b) in Fig. 3. Separately inspecting the employment behavior of perms and temps across recessions, we observe a larger loss of permanent employment levels in the COVID Recession than in the Great Recession one year into the recession; see, respectively, the center and right columns of panel (b) in Fig. 3. This larger loss of perm employment during COVID-19 than during the Great Recession persists at least up to our last observation—i.e., five quarters into the recession. In contrast, the loss of employment in temporary contracts—while larger than the contemporaneous loss in permanent contracts—is smaller during COVID-19 than in the Great Recession, except for the deep drop in 2020Q2, which shows an employment loss of 0.03 as opposed to the loss of 0.02 in the analogous 2008Q2 of the Great Recession. Then, three quarters into the recession, the loss of temp employment in the economy is about similar in both recession. Finally, four quarters (and further) into the recession the loss of temp employment during COVID-19 is smaller than that of the Great Recession. Interestingly, even though the loss in employment in the total economy is still mostly accounted by temps in 2020Q3 (approximately two-thirds of the total drop in employment), there is a tendency to symmetry, and by 2021Q1, perm and temps almost split their contribution to the total employment loss in half. Hence, not only the employment loss is smaller during COVID-19 than in the Great Recession, but also it seems it becomes more symmetric over time across temps and perms in the COVID-19 than one year into the Great Recession.

**Hours per worker** The behavior of (weekly) hours per worker also differs greatly across recessions; see panel (c) in Fig. 3. Whereas hours per worker do not respond in a significant manner during the Great Recession, hours per worker largely drop by 6.5 in 2020Q2—a 20 percent reduction below trend—during COVID-19 and seem relatively steady below trend, between 1.5 and 2 less hours since then. The drop in hours per permanent worker is a little over 7 hours and by temporary workers a little less than 5.5 hours. Subsequently, hours per worker recover less for permanent than temporary workers at a respective loss of 2.5 and 1 in 2021Q1. O

Overall, even though the employment loss for the total economy in 2021Q1 is approximately sixty percent lower than that of Great Recession at the same stage of the recession—i.e., one year into the recession, the large drop in hours per worker during COVID-19– makes the behavior of total aggregate hours across the two recessions extraordinarily similar—after the initial COVID-19 shock. Isolating the effects of perms and temps, we find substantial differences across recessions. During the first stages of the Great Recession (2008Q1-2009Q1), the loss of aggregate hours is almost entirely accounted for a by a loss of temp employment. In contrast, during COVID-19 and for stage of the recession (2020Q1–2021Q1), the loss of aggregate hours is a composite of losses from perms and temps. Although it is temps that suffer the largest losses in employment—accounting for two-thirds of the total losses in 2020Q1, perms account for one-third of the employment losses. These employment losses become more symmetric over time up to point where in 2021Q1 the contribution of the loss in employment in the total economy is almost equally explained by perms and temps. Further, the COVID-19 response comes with a higher adjustment in hours per worker for the perms, relative to the temps. This implies that overall, the effect of the recession in terms of aggregate hours is actually larger for perms than for temps throughout the currently observable quarters of the COVID-19 Recession though with a tendency to more symmetry over time.

### The role of ERTEs: an exploratory analysis

Although we cannot pinpoint the specific origins of the large differences between the labor market behavior during COVID-19 and the Great Recession that we document, it is reasonable to suspect that ERTEs may have had impact. However, identifying the isolated effects of ERTEs is problematic given the package of alternative policy measures that was implemented almost simultaneously; see our discussion in Sect. 2.3. In this context, in order to explore the potential role of the ERTEs, we simply re-conduct our analysis removing from our sample of employed individuals the population that reports being under ERTEs.^22^ Clearly, this experiment is not identical to a counterfactual scenario that shows the behavior of the Spanish labor market had the ERTEs not been implemented. The reason is that we do not know whether the individuals with ERTEs would have lost their job without the implementation of the ERTEs. Hence, our experiment simply contributes to highlight the differential employment (and hours per worker) of the actual economy (benchmark sample) with ERTEs versus an alternative view of the same economy where the individuals under ERTE are not considered employed (i.e., a restricted sample that excludes individuals with ERTEs from the employed population).

Our results are in Fig. 3. The differences between the behavior of employment in our benchmark sample with ERTEs (solid orange line) and our restricted sample without ERTEs (dashed yellow line) are substantial. In terms of employment, if we do not count ERTEs as employment, the economy suffers approximately twice the employment losses in 2020Q2; see panel (b) in Fig. 3. Further, decomposing the effects of ERTEs into perms and temps, we find that the ERTEs have largely cushioned permanent employment. Precisely, the loss of employment for perm workers is more than three times larger for the sample without ERTEs than for the benchmark sample with ERTEs. The ERTEs also helped cushion temp employment, but to a much lesser extent. Indeed, for the sample economy without ERTEs the employment losses are larger for the perms (more than 6 percentage points relative to the working-age population), than the losses for the temps (approximately 5 percentage points relative to the working-age population).

In terms of hours per worker, note that employed individuals with an ERTE report zero hours of work. Further, recall that hours per worker are computed as the ratio between aggregate hours and employment. Hence, ERTEs can be a (mechanic) rationale for the observed drop in hours per worker as the individuals with ERTEs reduce aggregate hours (numerator) without reducing employment (denominator) since individuals with ERTEs while reporting zero hours remain officially employed. We can assess this hypothesis by constructing hours per worker for our restricted sample that does not consider individuals with ERTEs employed. We find that even for the part of the employed economy that is not subject to ERTEs, hours per worker drop; see panel (c) in Fig. 3. Indeed, the drop in hours per worker for this restricted sample accounts for approximately half of the total drop in hours per worker. That is, the ERTEs help explain the drop in hours per worker, but half of that drop is still unaccounted for. This insights on hours per worker apply for both the perms and the temps with a stronger drop—also without ERTEs—for the perms.

Therefore, our analysis shows that, in addition to preventing employment losses, the ERTEs help explain the drop in hours per worker. At the same time, our analysis shows that although the ERTEs help reduce employment losses, they do not fully mitigate them. That is, the ERTEs do not sustain a story whereby—without employment losses—the sole margin of adjustment is hours per worker. In other words, the adjustment in hours per worker is not the only differential aspect of the COVID-19 Recession with respect to the Great Recession. During COVID-19, perm workers suffer large employment losses even with ERTEs. Further, the drop in hours per worker cannot be fully attributed to ERTEs since hours per worker substantially drop for individuals without ERTEs. Part of this drop in hours per worker that we document for the sample economy without ERTEs—in both perms and temps—could be partly explained by the 2012 reform that increased the ability to re-negotiate at the firm level the collective sector-level agreements regarding hours which increased the flexibility of hours per worker (Doménech et al. 2018). It is likely that the possibility to adjust hours per worker for those individuals without ERTEs also prevented further employment losses in both perms and temps. In the Great Recession, which preceded the 2012 reform, it was harder for firms to adjust hours per worker. This implies that in order to reduce labor input, the economy had to endure employment losses during the Great Recession, in particular for temps on the onset of the recession—a typical dual labor market outcome. In contrast, during the COVID-19, we find employment losses even for the perms. However, the duality of the labor market during COVID-19 is still present in the same direction as in the Great Recession in the sense that temps show approximately five times higher employment losses than perms in *relative* terms as percentage deviations from their trends, respectively, a loss of 25% of employment for temps and 5% for perms; see Fig. 4. Interestingly, we find that this duality result is in large part sustained by the ERTEs since the restricted sample without ERTEs drops the differential factor of employment losses between temps and temps from five to two. Precisely, without ERTEs perms show a loss of employment of approximately 15% below trend and temps show a loss of employment of approximately 30% in 2020Q2. In terms of hours per worker, we find that with or without the ERTEs, the magnitudes in the drop of hours across perms and temps are similar. In general, the effects of the ERTEs are substantially reduced from 2020Q3 and onward.

### An analysis of the joint dynamics of perms and temps hours

**Fig. 5:**
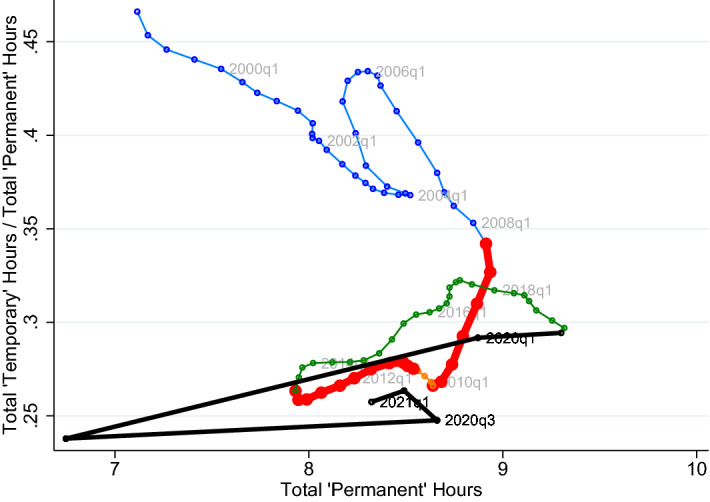
Joint dynamics of temp and perm hours. *Notes:* We use aggregate hours of perms and temps in the private sector. Our measure of aggregate hours is normalized by the working age population and are interpreted as the number of weekly hours devoted to production, per working-age person in Spain. To improve the visualization, we have slightly smoothed the evolution of the temps to perms ratio, up to 2019Q4; to fully capture the dynamics in the Covid-19 Recession, we have left these without smoothing. The quarters in the pre-2008 recession era are marked in blue, the twin recessions of the Great Recession in thicker red (the short intervening period in orange), the post-Great Recession recovery in green and Covid-19 in thicker black. We exclude construction from the private sector, and conduct a by industry analysis in Sect. 6.1 (color figure online)

The differential response of both employment and hours per worker across recessions that we document implies that focusing solely on employment to understand the labor market behavior provides a partial view of the actual adjustment of the labor input. With the benefit of hindsight, we now study more closely the behavior of aggregate hours which encompasses both employment and hours per worker dynamics. In particular, we study the complete path of the dual labor market behavior in terms of aggregate hours during the Great Recession and compare it with that of the ongoing COVID-19 Recession. We focus on the separate response of aggregate hours by perms and temps.^23^ In particular, to document the changing hours balance between perms and temps we plot the ratio of the aggregate hours of temps to those of perms (vertical axis) against the aggregate hours of perms (horizontal axis); see Fig. 5. In this manner, a vertical movement means that temps’ aggregate hours bear the burden of the adjustment (increasing if moving up, decreasing if moving down), while a horizontal movement means the adjustment is equally shared among the two types of workers. Hence, if permanent workers are the only ones adjusting, then there is a diagonal movement northwest (if perms’ hours decrease) or southeast (if perms’ hours increase). A diagonal southwest move indicates that both perms and temps adjust downward, but temps are disproportionately affected. Finally, a northeast move would indicate both perm and temp hours increase, but the hours increase relatively more for temporary workers. We conduct this exercise by industry.

**The Great Recession: a** *J* **-pattern and clockwise loop** First, early in the Great Recession—approximately the first six quarters that follow 2008Q1, we find that the ratio of temp hours to perm hours declines noticeably for the private sector; see panel (a) in Fig. 5. This occurs at the same time that perm hours adjust much more slowly. The combination of these two phenomena shows a relatively steep fall in our scatter plot (thicker red line after thin blue line), which implies that most of the adjustment comes from temp hours early in the Great Recession; see also Sect. 3.2. Beyond this, in the second part of the Great Recession (from 2010 to 2013Q2)—a period that concatenates the 2008 financial crisis with the European sovereign debt crisis, we find that the decline in the temp-to-perm hours ratio slows down, while permanent hours drop throughout which flips the margin of adjustment from temps to perms. As a result, we find a *J*-pattern (or -pattern) for the Great Recession in Spain from 2008 to 2014 in the private sector.

Then, in the initial phases of the recovery—from 2013 to 2016—we see a response (green line segment) that generally lies above that of the Great Recession; see panel (a) in Fig. 5. As hours increase in a recovering sector, the ratio of temporary to permanent hours grows: Aggregate hours increase by utilizing relatively more hours from temps. Moreover, for most market sectors, in panels (b)–(e), the ratio of temp to perm hours is larger in the recovery, for each level of perm hours, than it was in the recession before. This implies that the evolution of temp hours and perm hours during the Great Recession and its recovery—i.e., piecing together the red and green lines in Fig. 5—is well summarized by a *clockwise loop*, clearly recognizable in the private industries.

**COVID-19 Recession** The initial response up to (and including) the second quarter of 2020 is a sharp leftward and somewhat less strong downward move; see black line panel (a) in Fig. 5. That is, initially, the COVID-19 Recession implies a massive loss of permanent hours which is accompanied by a more moderate decline in the ratio of temp to perm hours. This is consistent with our previous results—indeed temp hours drop by a lesser magnitude than perm hours with respect to their associated trends; see Sect. 3.2. Further, note that the change in permanent hours in 2020Q2 alone is larger than the entire drop in permanent hours during the Great Recession. Indeed, in the private sector, permanent hours in 2020Q2 mark a twenty-first century lowest. At the same time, the drop in the ratio of temp to perm hours is clearly visible between 2020Q1 and 2020Q2, but smaller than the total drop of this ratio through the entire Great Recession.

Clearly, the COVID-19 Recession contrasts greatly with what occurred early in the Great Recession where the margin of adjustment was initially (almost exclusively) born by temps. This is shared across most sectors; see panels (b)–(e) in Fig. 5. We see that the response for Hospitality, Retail, and Transport (HRT), as expected, is the strongest of all sectors in the COVID Recession; the construction sector on the other hand responds more weakly than it did in the Great Recession. Interestingly, in many sectors, the angle of black line covering the initial impact of COVID retraces the direction travelled during the post-2013 recovery closely (i.e., crossing the green curve somewhere around 2013 but, of course, extending further to the bottom right).

Finally, note that the partial recovery in the third quarter of 2020 comes with a sharp right-ward, near-flat move across almost all sectors: A substantial part of the earlier loss of permanent hours is recovered, *but not completely*, while the temp-to-perm hour ratio also does not fully recover. In particular, it does not appear to be the case that the end of the lockdown has necessarily set the response in the temp to perm ratio relative to permanent hours neither back to the start in 2019Q4, nor put it on a similar dynamic track as occurred the Great Recession, with dominant total temp hours losses. After the lockdown ended, still relatively more use is being made of the permanent margin than at the beginning of the recession before, and that this shared by many sectors. The partial recovery after the lockdown, nevertheless, leaves space that, if, e.g., the ERTEs were to be phased out, the economy could return to a point that effectively lies on a “Great Recession”-like *J*-shaped curve in Fig. 5 originating from the 2019Q4 data point.

## A production model with temp and perm hours

In this section, we lay out a model that can give rise to the above clockwise adjustment pattern in the simplest way possible, and use it as a device to discuss the patterns in Fig. 5 for both the Great Recession and the COVID Recession. By construction, this model abstracts from many dynamic considerations—as such it cannot capture the full richness of distortions associated with duality in the labor market. However, it (re)emphasizes elements present already, e.g., in Dolado et al. (2002) that are relevant in light of the above analysis, and argues these should be taken seriously in further dynamic investigations. We discuss this further at the end of this section.

Consider a constant elasticity of substitution production function that relies on the aggregate hours of permanent workers, $H_P$, and those of temps, $H_T$, following Dolado et al. (2002):^24^

2 $$\begin{aligned} P Y=P A K^{\alpha }H^{1-\alpha }, \text { with } H=\Big (\eta _P H_P^\frac{\sigma -1}{\sigma } + \eta _T H_T^\frac{\sigma -1}{\sigma }\Big )^\frac{\sigma }{\sigma -1}, \end{aligned}$$

where $\sigma$ is the elasticity of substitution between permanent workers and temps and $\eta _T, \eta _P$ are weights that allow us to match the shares of temporary hours vs permanent hours in production. Define $p=P A$, so that we can capture both shocks to *P* (demand) and *A* (technology) in one variable.^25^

Fix hourly wages for temps, $w_T$, and perms, $w_P$. Workers are hired competitively and employed in a representative firm with production function (2), while capital *K* is fixed throughout the analysis. We consider only a one-period static decision making, with prices taken as given. To build our argument, we introduce one friction: Firms, however, have inherited a stock of temporary and permanent workers and face a firing cost on the permanent workers. As in panel (c) in Fig. 2, we see that in the Great Recession an hours reduction implies an employment reduction (as hours per worker are approximately constant); we therefore phrase the firing cost as cost per unit reduction $\tau$ of $H_P$ from $H_P^0$.^26^

The maximization problem for the firm thus is to maximize

3 $$\begin{aligned} p K^{\alpha }\Big (\eta _P H_P^\frac{\sigma -1}{\sigma } + \eta _T H_T^\frac{\sigma -1}{\sigma }\Big )^\frac{(1-\alpha ) \sigma }{\sigma -1}- w_T H_T - w_P (H_P +\tau \max \{H_P^0-H_P, 0\}) . \end{aligned}$$

The associated first-order condition with respect to $H_T$ equals

4 $$\begin{aligned} w_T=p \ \eta _T (1-\alpha )K^{\alpha } \Big (\frac{H_T}{H_P}\Big )^{-\frac{1}{\sigma }} \bigg (\eta _P + \eta _T \Big (\frac{H_T}{H_P}\Big )^{\frac{\sigma -1}{\sigma }}\bigg )^{\frac{1-\sigma \alpha }{\sigma -1}} \cdot H_P^{-\alpha }. \end{aligned}$$

The actual choice of $H_T$ depends on whether and how permanent hours $H_P$ adjust as well, which we cover in the next result:

### Result 1

Given $H_P^0$, there exists a unique ${\underline{p}}, {\overline{p}}$, with ${\underline{p}}(H_P^0)=$ $p\le {\overline{p}}(H_P^0)$

1. for any $p\le {\underline{p}}(H_P^0)$, $H_T$ and $H_P$ are such that they satisfy FOC (4) and the ratio of $\frac{H_T}{H_P}$ equals $\underline{\frac{H_T}{H_P}}$, where
  5 $$\begin{aligned} \underline{\frac{H_T}{H_P}}=\left( \frac{\eta _T (w_P(1-\tau ))}{\eta _P w_T}\right) ^\sigma \end{aligned}$$
  We can calculate that
  6 $$\begin{aligned} {\underline{p}}(H_P^0)=(H_P^0)^{\alpha }\cdot \frac{w_P(1-\tau )}{1-\alpha } \eta _P^{\frac{\sigma (1-\alpha )}{1-\sigma }} \left( 1+ \left( \frac{\eta _T}{\eta _P}\right) ^{\sigma } \left( \frac{w_T}{w_P(1-\tau )}\right) ^{1-\sigma }\right) ^{\frac{1-\alpha \sigma }{1-\sigma }} \end{aligned}$$
2. for any $p\ge {\overline{p}}(H_P^0)$, $H_T$ and $H_P$ are such that they satisfy FOC (4) and the ratio of $\frac{H_T}{H_P}$ equals $\overline{\frac{H_T}{H_P}}$, where
  7 $$\begin{aligned} \overline{\frac{H_T}{H_P}}=\left( \frac{\eta _T w_P}{\eta _P w_T}\right) ^\sigma \end{aligned}$$
  We can calculate that
  8 $$\begin{aligned} {\overline{p}}(H_P^0)=(H_P^0)^{\alpha }\cdot \frac{w_P}{1-\alpha } \eta _P^{\frac{\sigma (1-\alpha )}{1-\sigma }} \left( 1+ \left( \frac{\eta _T}{\eta _P}\right) ^{\sigma } \left( \frac{w_T}{w_P}\right) ^{1-\sigma }\right) ^{\frac{1-\alpha \sigma }{1-\sigma }}, \end{aligned}$$
  and it follows that for $\tau >0$, it holds that ${\underline{p}}(H_P^0) < {\overline{p}}(H_P^0)$, for any $\sigma \ge 0$ (given $0<\alpha <1$).
3. for any ${\underline{p}}(H_P^0) \le p\le {\overline{p}}(H_P^0)$, $H_P=H_P^0$ and $H_T$ satisfies first-order condition (4) and
  9 $$\begin{aligned} \left( \frac{\eta _T w_P(1-\tau )}{\eta _P w_T}\right) ^\sigma \le {\frac{H_T}{H_P^0}}\le \left( \frac{\eta _T w_P}{\eta _P w_T}\right) ^\sigma \end{aligned}$$

**Fig. 6:**
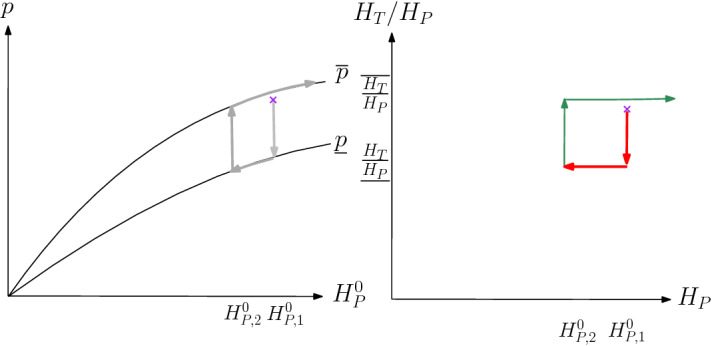
Evolution of productivity/demand *p*, perm hours $H_P$, and temp-to-perm hours ratio $H_T/H_P$

With Result 1 in hand, it is easy to characterize what will happen when *p* declines substantially then recovers, in a sequence of repeated instances of the above static problem, without intertemporal links in the objective function, other than through the evolution of $H_P^0$. One interpretation of the exercise would be profit maximization by extremely myopic firms. Thus, the representative firm would, given wages, maximize inputs $H_T$ and $H_P$, where the latter becomes the next period’s $H_P^0$.

Given this, we can work toward characterizing the dynamics, as a sequence of static maximizations, given evolving *p* and $H_P^0$. First, note that ${\underline{p}}(H_P^0)$ and ${\overline{p}}(H_P^0)$ are concave functions of $H_P^0$, as in the left panel of Fig. 6. Suppose that we start at the purple x, and the economy will enter in recession (*p* decreases). At first as *p* drops, we are in the third case of result 1: Temporary workers are shed, and $H_T/H_P^0$ drops but $H_P^0$ remains unchanged, until *p* hits the ${\underline{p}}$ frontier. Further decreases in *p* now trigger a proportional decrease in $H_T$ and $H_P$, keeping the ratio constant at $\underline{\frac{H_T}{H_P}}$. In Fig. 6, this means a move down along the ${\underline{p}}$ in the left panel, and a horizontal move in the right panel. When the recovery starts and *p* starts to increase again, at first it is strictly better to hire only temporary workers. The marginal product of temporary workers has gone up, at the previous period’s level of $H_T$ and $H_P$; however, not so many temps have been hired yet that the MRS $(MPL_T/MPL_P)$ has gone down to relative wage ratio $\frac{w_T}{w_P}$, where increasing permanent hours would become beneficial. (Subscript *T* points to the temporary hour wage and MPL, *P* to the permanent hour analogues.) As long as *p* rises, but stays in the range associated with case 3 in result 1, $H_P^0$ stays constant. Finally, when ${\overline{p}}$ is hit, an increase in p, triggers an increase in $H_P^0$, to keep $H_T/H_P$ at $\overline{H_T/H_P}$.

Thus, firing costs in this extremely simple and intuitive setting, gives rather straightforwardly rise to clockwise loops, as we saw it in the data. In contrast, ERTEs allow costless adjustment in both permanent and temporary hours: A corresponding case in our simple theory would be the firing cost for permanent workers dropping to zero. Then the area of the clockwise loop will collapse, and adjustment will take place according to (7) and (8), with the latter yielding $H_P$ according to $p=(H_P)^{\alpha }\cdot \frac{w_P}{1-\alpha } \eta _P^{\frac{\sigma (1-\alpha )}{1-\sigma }} \left( 1+ \left( \frac{\eta _T}{\eta _P}\right) ^{\sigma } \left( \frac{w_T}{w_P}\right) ^{1-\sigma }\right) ^{\frac{1-\alpha \sigma }{1-\sigma }}$. Thus, a reduction in production following a negative shock to *p* will now follow the upper curve, not the lower curve, in Fig. 6 and re-trace any previous expansion (when *p* was previously growing), consistent with the retracing we observe in the COVID-19 Recession part of Fig. 5 in the previous section.^27^ A renewed recovery in *p* would once again trace out this curve.^28^

Of course, the above discussion uses a simple model to intuitively illustrate the economic mechanism behind the data patterns in Sect. 6.1. A more in-depth investigation could incorporate this in a state-of-the-art properly dynamic equilibrium model.^29^ A fully dynamic model could incorporate the opportunity-cost motive along the lines that we mentioned in the previous section: In a declining industry, a permanent worker who is not fired today may very well be fired tomorrow (with the firm incurring the firing cost in any case, though possibly discounted). More generally, firms face an intertemporal trade-off between having a stable workforce (that allows investment in potentially specialized human capital, and less hiring costs) and adjustment flexibility to negative shocks that we have clearly abstracted from.^30^

Note further that wages have been held constant throughout this exercise. As such, it appears difficult to use the clockwise loop patterns by themselves to estimate the elasticity of substitution between permanent and temporary hours in production. While the difference between ${\underline{p}}(H_P^0)$ and ${\overline{p}}(H_P^0)$ responds to $\sigma$ and $\tau$, the extent of *p* changes affects how much the $H_P$ moves horizontally. Let alone, that any additional heterogeneity (e.g., as mentioned in footnote 29) also shapes the loop. Again, this points to the need for fully specified, dynamic models of the labor market [building, e.g., on Bentolila et al. (2012a), among others] to help understand fully the dual labor market over the cycle.

## Discussion

Thus, in the previous section, we have discussed that a simple model with an integrated production of temporary and permanent workers and costly firing of permanent workers can intuitively capture the clockwise dynamics that we documented for the Great Recession and its recovery in Sect. 3. Further, as we also documented, the initial drop in temp hours with relatively little change in perm hours in the Great Recession does not apply to the COVID-19 so far; instead, there is an immediate strong adjustment of permanent hours. Again, the simple model, now with costless adjustment of labor, can help provide a rationalization for this.

**Large adjustments across recessions and their implications** The flexibility to adjust hours, inherent to ERTE/short-time work policy, could help to distribute the impact of the COVID shock across the labor market, not just the parts where temporary employment dominates. The nature of ERTEs across both temporary and open-ended contracts suggests a possible direction in which adjustment would occur without firing restrictions. We can see a suggestive relation for this when we put the “deviation-from-trend” measurement of Sect. 3.2, together with the subsequent analysis in Sects. 6.1–4, in the form of two graphs.

**Fig. 7:**
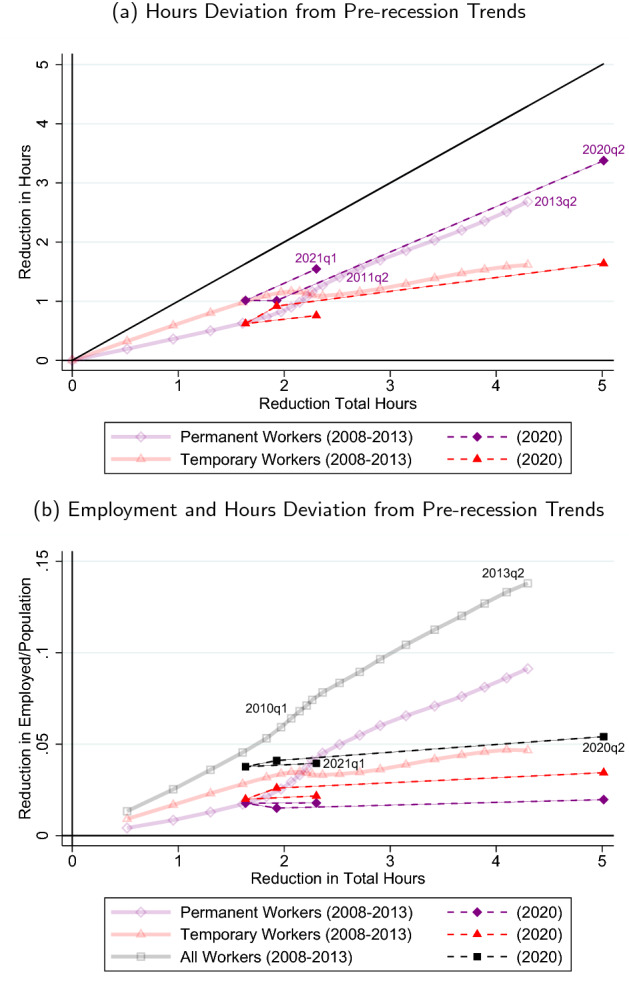
Hours and employment deviations from pre-recession trends: temps and perms

In Fig. 7, we plot a smoothed version of the total economy hours deviation from the pre-recession trend on the x-axis and set it against the deviations of total, temporary, and permanent hours, for both recessions in Fig. 7a and employment in Fig. 7b. For the lighter (more transparent) lines corresponding to the Great Recession, each quarter these gaps relative to previous trend increased, so a rightward movement along markers also corresponds to the quarterly evolution of the variables in question.^31^ For the COVID Recession, we have labeled the quarters, starting by 2020Q2 at the very right (the biggest hours response), followed by a leftward move in 2020Q3, and ultimately a much smaller rightward move in 2021Q1.

Figure 7a illustrates that, in terms of accumulated deviation from pre-recession trend, the Great Recession, at the trough in 2013, had a loss in aggregate hours not too different from the second quarter in 2020 deviation (relative to the pre-2020 trend, here calculated from 2015 with minor impact). As our discussion in Sect. 6.1 indicates, the evolution of the relative importance of permanent vs temporary hours loss clearly contrasts between the two recessions. At the beginning of the Great Recession, more than two-thirds of the losses were borne by temporary workers, with the relative importance of temporary vs. permanent hours reduction only switching 1.5 year into the Great Recession—very different from the pattern in the first quarters of the COVID Recession. Only at the end of the Great Recession, after the permanent losses in the later years of it, the relative division of the losses between permanent and temporary hours has become roughly similar, though (even with the larger aggregate hours loss) the ratio is still larger from peak to trough of the Great Recession.

Figure 7b then compares the reduction (deviation from trend) in temporary and permanent *employment*, on the y-axis, to the aggregate hours deviation, again on the x-axis. For the Great Recession, we see that the reduction in workers follows closely the shape of the hours pattern over time. The reduction in employment during the COVID Recession is much less in comparison *given the enormous amount of hours reduction*. Note that even though the level of temporary employment losses is higher than permanent losses in COVID times, it lies strictly below its corresponding curve for the Great Recession, leaving suggestive room for ERTEs to keep some temporary employment going that in its absence would have been destroyed. Relative to the aggregate hours loss, the destruction of permanent employment in 2020Q2 has been much less than accumulated in the Great Recession. With the partial recovery of the economy in the next two quarters (2020Q3-Q4), the loss of permanent employment in 2020Q4 remains below or equal to curve of the Great Recession, while in 2021Q1 aggregate hours drop without additional meaningful adverse effects on permanent (or for that matter, temporary) employment.

While the temp and perm employment losses are low relative to the aggregate hours lost in the economy, they are *large* in the first all-recession quarter, 2020Q2, when compared to the first quarters of the Great Recession (a very different comparison, mostly covered in Fig. 3). The relative size of temporary and permanent employment losses early in COVID times has a ballpark ratio of around 2-to-1 that mimics the ratio early in the Great Recession (when aggregate hours losses were up to 1.5-2 hours).^32^ After the lockdown ended, the hours losses diminished, and employment losses relative to aggregate hours losses became more in line the corresponding patterns the Great Recession (i.e., with less distance between the COVID-Recession and Great-Recession data points corresponding to aggregate hours losses around 2), it remains noteworthy that temporary employment losses are relatively lower (with a noticeable gap) in the COVID Recession than in the Great Recession for similar levels of aggregate hours losses.

Figure 7 leaves room for the multiple dimensions of the ERTE/short-time work policy. By construction, this policy will help avoid dismissals of workers in permanent *and* temporary contracts, an avoidance that can be rationalized by the nature of the COVID shock. However, the impact of the policy can go beyond this: With its strength depending on the elasticity of substitution $\sigma$, it allows a less distorted reduction in employment and hours, which (by virtue of being closer to the unconstrained choice) further could save viable production overall, relative to what would have been in the absence of ERTEs. Indeed, the use of ERTEs allows firms to bypass the effective ‘tax’ wedge in adjustment of the two types of labor, and could lead to lower misallocation at time of a serious negative shock.^33^ More generally, with firing costs distorting the type of workers that are hired and dismissed, thereby distorting average productivity, this points to a further discussion (outside the scope of this paper) on the benefits of introducing permanent short-time work regulation, beyond the COVID-19 Recession.

## Robustness and further context

First, we explore the robustness of some our empirical results. In particular, we focus on the behavior of aggregate hours across industries in Sect. 6.1. Second, we briefly explore two further dimensions by which the Great Recession and the COVID-19 Recession differ including business closures (and formation) in Sect. 6.2 and parental labor supply in Sect. 6.3.

### A cross-industry analysis

**Fig. 8:**
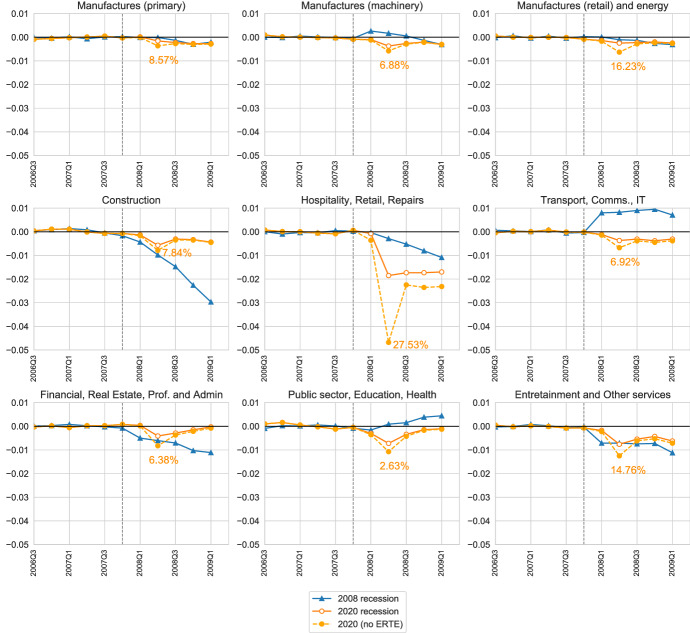
*Absolute* response of employment by industry in the Great Recession and COVID-19. *Notes:* All variables are plotted as level deviations from a predicted trend from 1 that uses data up to the onset of recessions. The measuring and lines are equivalent from those in Fig. 3. We also annotate the percentage of workers under an ERTE in 2020Q2 in each plot

An aspect that reveals that Great Recession and the COVID-19 Recession are different in nature is the fact that each recession affected different industries more than others. Here, we explore how the heterogeneous response across industries differs by industry. First, we reconduct our empirical analysis in Sect. 3.2 by industry; see Fig. 8. We do this exercise to shed more light on the different nature of the two recessions. We focus on employment, for simplicity. We find, as expected, that the impact of the COVID-19 Recession was much more immediate and evident in all industries. In contrast, it took time for many industries to be noticeably affected in the Great Recession, while construction and real estate were immediately affected. That is, the Great Recession was mainly driven by the collapse of construction and its related industries, while the COVID-19 shock is more evenly shared. That being said, hospitality and retail industries have suffered a much larger impact than other industries, with an employment loss of 2% of the working-age population in the second quarter of 2020 alone. The effect of ERTEs is very unevenly split. We find that in 2020Q2, more than 27% of individuals working in hospitality and retail were subject to an ERTE. This share is much larger than any other industry—the second highest being manufactures of final goods (retail) with 16.23%. In contrast, only 2.63% of employed workers were under an ERTE in the public sector, and between 6% and 7% in the service industries which are more amiable to remote working arrangements: communications and IT and professional services. Finally, it is worth noting the similarity between the fall in construction (without ERTEs) during 2020Q2 and that seen in the Great Recession. Taken together with the large take-up of ERTEs in hospitality, this provides strong evidence that these short-term work policies have had a large impact on employment. More precisely, if remove the individuals with ERTEs from the employed population, we find that across most industries the ERTEs cut the employment losses by approximately by one half. At the same time, this implies that one half of the employment losses remain unexplained. These results mimic the aggregate results discussed in Sect. 3.3.

**Fig. 9:**
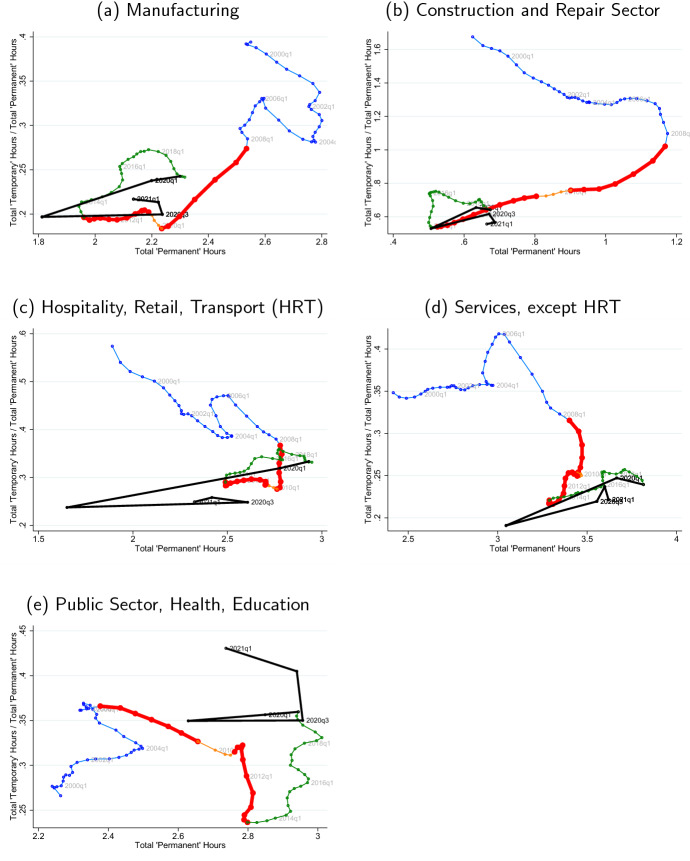
Joint dynamics of temp and perm hours: by industry. *Notes:* In panel (a), we exclude construction from the private sector. Aggregate hours are normalized by the working age population and are interpreted as the number of weekly hours devoted to production, per working-age person in Spain. To ease the visualization, the evolution of the temps to perms ratio is slightly smoothed, up to 2019Q4; to fully capture the dynamics in the Covid-19 Recession, we left these without smoothing. The quarters in the pre-2008 recession era are marked in blue, the twin recessions of the Great Recession in thicker red (the short intervening period in orange), the post-Great Recession recovery in green and Covid-19 in thicker black (color figure online)

Second, we reconduct our analysis of the dual market hours by industry in order to assess the robustness of the *J*-pattern (or -pattern) that we documented for the Great Recession in Spain from 2008 to 2014 in the private sector in Sect. 3.4. We find that is also the case by industry, somewhat less pronounced in Manufacturing and in Construction and Repair (see, respectively, panels (a) and (b) in Fig. 9) and somewhat steeper effects (almost a vertical line) in Hospitality, Retail, and Transport (HRT) and in Services (see, respectively, panels (c) and (d) in Fig. 9).^34^ In contrast to the market sector, the public sector first sees an increase of perm hours and does not shed temp hours as fast as other sectors; see panel (e) in Fig. 9. However, in the second half of the recession there is a substantial drop in temp hours while perm hours stay stable. This suggests the public sector acted as a reservoir of employment early in the recession, but after the 2011 Eurozone crisis it was forced to adjust its workforce, and did so in the same way as other sectors started to cut hours at the beginning of the Great Recession, by cutting out temps. Further, focusing on the initial phases of the recovery—from 2013 to 2016, we note that the clockwise loop appears most pronounced in stable or declining sectors, where the onset of recovery involves proportionally higher temp hours. On the other hand, structurally growing sectors such as Services start adding perm hours relatively early in the recovery, which flattens or shrinks the loop (see panel (d) in Fig. 9. Finally, again, the public sector behaves differently: Early in the recovery, it grew by expanding temp and perm positions nearly proportionally, only to start adding mostly temp hours from 2015 onward.

**Table 1:** Aggregate hours and relative temp/perm hours across recessions, by industry

Industry	2008Q2	2013Q2	Rel diff. (%)	2019Q4	2021Q1	Rel. diff (%)
*Aggregate hours*						
Manufacturing	3.46	2.47	$-$ 29	2.87	2.69	$-$ 7
Construction & Repairs	2.51	0.83	$-$ 67	1.11	1.04	$-$ 7
HRT (Hosp., Retail, Transport)	3.97	3.28	$-$ 17	3.94	2.89	$-$ 27
FIRE, IT & Communication	1.41	1.26	$-$ 10	1.57	1.48	$-$ 6
Professional & aux. services	2.15	1.87	$-$ 13	2.21	2.17	$-$ 2
Public Sector, Educ, Health	3.61	3.77	4	4.19	4.13	$-$ 1
Entertainment & other services	1.14	1.01	$-$ 11	1.03	0.87	$-$ 16
All	18.26	14.51	$-$ 21	16.93	15.26	$-$ 10
*Ratio total temp hrs/total perm hrs*						
Manufacturing	0.27	0.18	$-$ 32	0.24	0.21	$-$ 15
Construction & Repairs	1.01	0.53	$-$ 47	0.63	0.53	$-$ 15
HRT (Hosp., Retail, Transport)	0.35	0.22	$-$ 35	0.32	0.22	$-$ 31
FIRE, IT & Communication	0.26	0.21	$-$ 19	0.21	0.21	1
Professional & aux. services	0.26	0.19	$-$ 27	0.22	0.19	$-$ 13
Public Sector, Educ, Health	0.35	0.41	17	0.35	0.41	15
Entertainment & other services	0.43	0.23	$-$ 46	0.31	0.23	$-$ 24
All	0.41	0.24	$-$ 41	0.31	0.29	$-$ 8

Aggregate hours normalized by working-age population (as in the main text)

*FIRE* Financial Services, Insurance, and Real Estate. *Source*: INE

In Table 1, we summarize the *overall* hours change and the change in the relative ratio of total temp hours to perm hours, per industry, across the entire Great Recession and COVID-19 Recession, up to 2021Q1 (with partial post-lockdown recovery). The aggregate hours adjustments show the expected heterogeneity across sectors with higher losses for Construction in the Great Recession, and for Hospitality, Retail, and Transport for the COVID-19 Recession. But in terms of adjustment in the ratio of total temporary to permanent hours, note that the COVID-19 Recession comes with (often much) smaller losses across *all* sectors (besides the Public, Education, and Health Sector) than the Great Recession. Given the previous discussion, this is even more noteworthy: Early in the Great Recession, when aggregate hours losses were lower, the drop in the ratio of temp-to-perm hours was considerably larger than the drops reported here corresponding to the entire duration of the recession. Relative to the Great Recession, there still is plenty of adjustment done using the perm hours margin in 2021Q1, across all sectors.

### Business closures and business formation

It can be that adjustment in permanent or temporary hours (or both) is driven by firm closures.

**Fig. 10:**
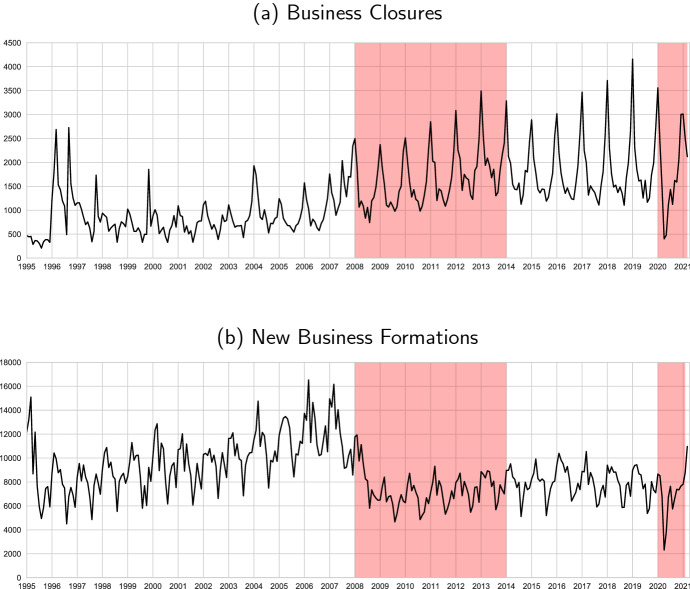
Business closures and new formations during the Great Recession and COVID-19. *Notes:* Business closures (“sociedades mercantiles disueltas”) and new business formations (“Sociedades mercantiles constituí­das") as reported by the Spanish Trade Bureau (Registro Mercantil). Source: https://www.ine.es/dyngs/INEbase/es/operacion.htm?c=Estadistica_C&cid=1254736177026&menu=ultiDatos&idp=1254735576550INE

However, business closures in Spain during the COVID-19 Recession do not increase; see panel (a) in Fig. 10. This is in contrast to the increase in business shut downs at the onset of the Great Recession and its aftermath. Given that firm closures in the COVID-19 Recession are lower than in the Great Recession, we can speculate that ERTEs may play a role in firm survival by lowering the operational costs of businesses—allowing firms to stay afloat—with a contractual arrangement that allows for a reduction of hours without expensive layoffs of permanent workers. Interestingly, the number of closures actually seems to decrease during COVID-19, which suggests that the ERTEs could also help the survival of firms that otherwise would close even without the COVID-19 crisis; a potential productivity effect/channel that we think deserves further exploration.

Further, one year into the COVID-19 Recession, the formation of new businesses shows a substantial increase from a monthly average of 8000 new businesses formed during the years preceding the COVID-19 recession up to approximately 11,000 new businesses formed in the first months of 2021—a record high since the onset of the Great Recession; see panel (b) in Fig. 10. This feature may help to explain some of the employment recovery that we start to see since 2020Q3.

Given these patterns, one may also worry that be that the overall permanent hour reduction in the second leg of the Great Recession is driven by firm closure—thus, the shift from temporary dismissals to permanent dismissals is not due to within-firm worker-type portfolio decisions, but rather across firms, involving firm exit. We have checked this in the social security data: For those permanent workers who work in firms for which we can see at least three other permanent workers in the data (hence, we are confining ourselves to reasonably large firms), roughly 25% of employment loss is linked to firm exit in 2011–2012, somewhat higher than in other periods. However, the vast majority of permanent dismissals of workers in this type of firms, in the second half of the Great Recession, occur while the firm continues to operate, suggesting an important role for intra-firm adjustment in terms of permanent hours—all while acknowledging that firm entry and exit patterns and their difference across both recessions carry significant interest.

**Fig. 11:**
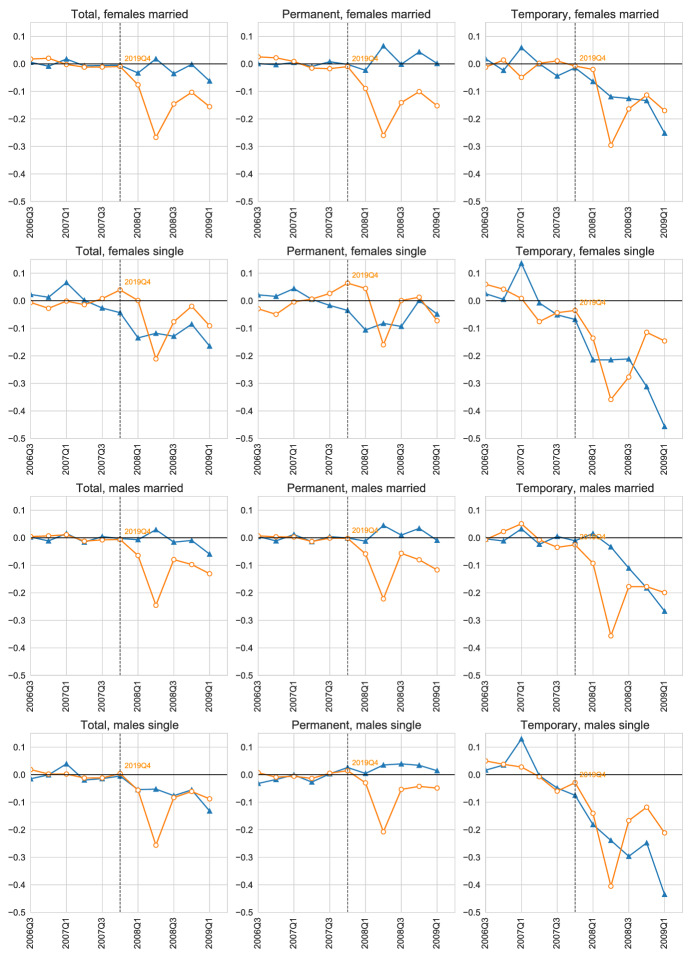
*Relative* response of aggregate hours by gender and marital status in the Great Recession and COVID-19, prime-age adults. *Notes:* Aggregate hours of married females means the sum of all hours worked by married females in the labor market. All variables are plotted as *relative* (percentage) deviations from a predicted trend that uses data up to the onset of recessions. Note that we have shifted the Covid-19 Recession in time in order to normalize the onset of the Covid-19 Recession to that of the Great Recession. Prime-age adults defined between 30 and 50 years old.

### Parental labor supply

The COVID Recession has put additional pressure on parents’ (or indeed, any carer’s) time, unlike other recessions.^35^ The closing down of schools in the first national lockdown in particular meant that children needed to be cared for at home. Therefore, parents can be especially affected in their labor supply and potentially respond with a reduction in labor hours on the market.^36^ To get a sense of parental labor supply considerations, Fig. 11 displays the *relative* (percentage) deviations from trend of the aggregate hours. We do this separately by category, per gender $\times$ marital status, and separately for perms and temps.^37^ We use marital status as an imperfect proxy for the presence of children in the household.^38^ If one of the main drivers in the drop in hours/employment is the need for parents to take care of children, we should observe hours falling more for married individuals—and if the load of care is not shared evenly among spouses (as it is the case in Spain), then married women should display the largest fall. We show the 2008 recession to offer a benchmark for comparison, as in Fig. 3.

On first approximation, we see that prime-aged permanent workers of all four (marital status $\times$ gender) categories experience a drop of over 20% in 2020Q2 relative to 2019Q4. A closer look shows that the aggregate hours of married females experience a deeper relative drop, closer to 30%. The drop of males is very similar for both married and singles. The relatively common behavior of aggregate hours in the COVID-19 Recession, across these four categories, limits the role of parental labor supply considerations in aggregate hours behavior of perms somewhat.

When we unpack these patterns, we observe that for perms, the hours per worker adjust very similarly across all four categories in the COVID-19 Recession (while in the Great Recession, there was very little action across all categories).^39^ Where we do observe differences is across the employment response: Employment in permanent contracts of married workers decreases more than that of their unmarried counterparts in permanent contracts during COVID-19. This is especially pronounced for married females and consistent with the parental labor supply story. Putting the hours per worker and employment responses together for aggregate hours, we see that the hours per worker behavior in COVID has a rather dominant effects. This dominant effect of hours per worker is behind the aforementioned large common component of aggregate hours behavior in Fig. 11 and leaves room for an important role of ERTEs. Nevertheless, the differences in employment behavior across marital status appear important and point to the COVID-19 Recession affecting married women in permanent contracts, especially badly. This is in line with, e.g., Farré et al. (2020), who find employment consequences for women in COVID-19 in addition to an unequal increase in household production responsibilities.

## Conclusion

The behavior of the dual labor market during COVID-19 differs substantially from the Great Recession. During the Great Recession, the initial response was mostly an employment loss for temps, whereas during COVID-19 employment and hours per worker drop with perms explaining more than half of the drop in aggregate hours. With hindsight, we also find that the flexibility that perms show at the onset of the COVID-19 Recession is in line with the accumulated response *five years* into the Great Recession, though with a very much smaller employment response. Further, we argue that behind the observed patterns of the dual labor market is more likely to be the effect of policy (e.g., ERTEs), rather than the different nature of the recession. Precisely, we show that while the industry impact is asymmetric and largely differs across recessions, the aggregate patterns stand within industry. In terms of what to expect from future recessions, our results suggest that, without ERTEs, the current degree of labor market flexibility—i.e., post-2012 reform—tends to make the dual labor market more symmetric across temps and perms but still falls short in homogenizing responses across perms and temps, in particular, in terms of employment.

We hope that our empirical description of the differential response of the dual market across recessions helps inform future analysis of the effects of policy, in particular, the effects of ERTE in the context of structural equilibrium models of the dual market and its distortions.

## Figures and Tables

**Fig. 12:**
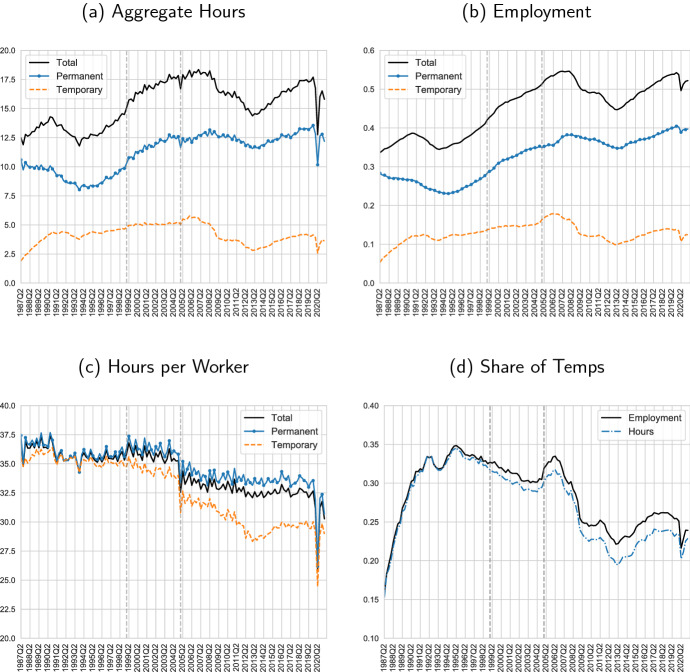
Hours and employment: total, perms, and temps, Spain 1987Q2–2021Q1. *Notes:* Aggregate hours and employment are divided by working age population. All variables are reported on a weekly basis. The vertical dashed lines correspond to the quarters in which the EPA survey was re-designed as described in the text

**Fig. 13:**
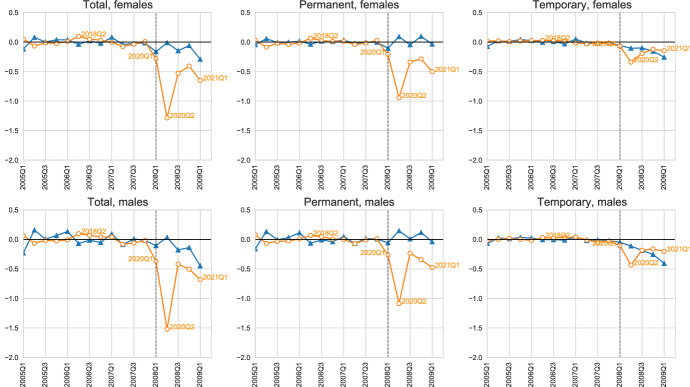
*Absolute* response of aggregate hours by gender in the Great Recession and COVID-19, prime-age adults. *Notes*: Aggregate hours of Females means the sum of all hours worked by females. All variables are plotted as *absolute* deviations from a predicted trend that uses data up to the onset of recessions. Note that we have shifted the Covid-19 Recession in time in order to normalize the onset of the Covid-19 Recession to that of the Great Recession. Prime-age adults are defined between 30 and 50 years old

**Fig. 14:**
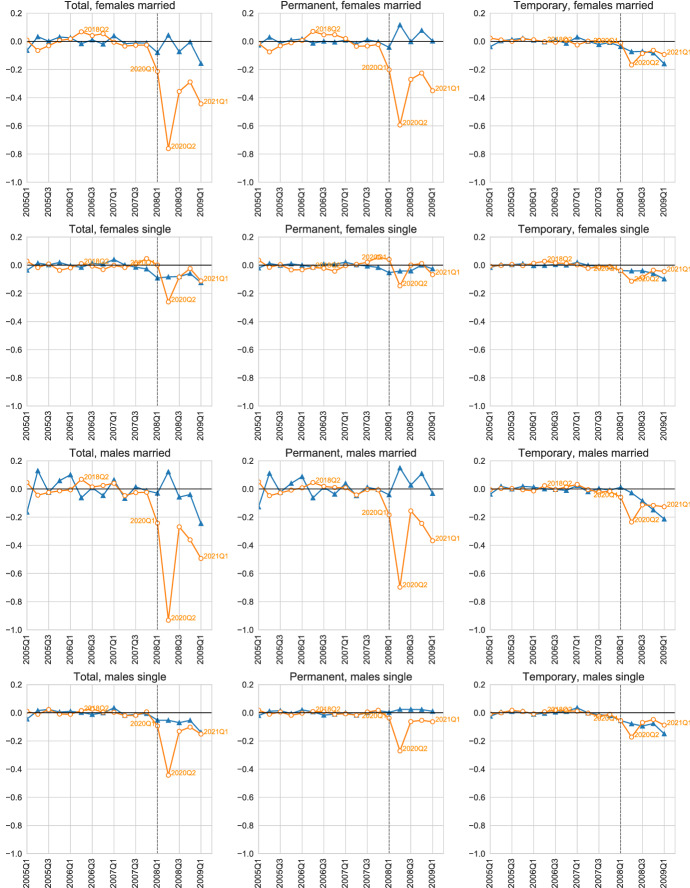
*Absolute* response of aggregate hours by gender and marital status in the Great Recession and COVID-19, prime-age adults. *Notes*: Aggregate hours of Married Females means the sum of all hours worked by married female. All variables are plotted as *absolute* deviations from a predicted trend that uses data up to the onset of recessions. Note that we have shifted the Covid-19 Recession in time in order to normalize the onset of the Covid-19 Recession to that of the Great Recession. Prime-age adults are de1ned between 30 and 50 years old

**Fig. 15:**
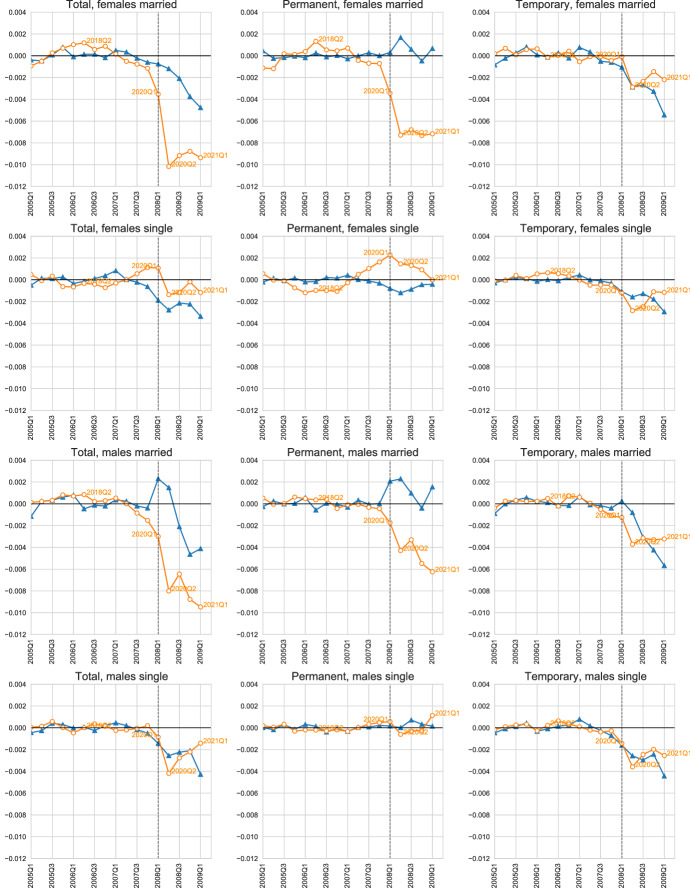
*Absolute* response of *employment* by gender and marital status in the Great Recession and COVID-19, prime-age adults. *Notes*: Aggregate hours of Married Females means the sum of all hours worked by married female. All variables are plotted as *absolute* deviations from a predicted trend that uses data up to the onset of recessions. Note that we have shifted the Covid-19 Recession in time in order to normalize the onset of the Covid-19 Recession to that of the Great Recession. Prime-age adults are de1ned between 30 and 50 years old

**Fig. 16:**
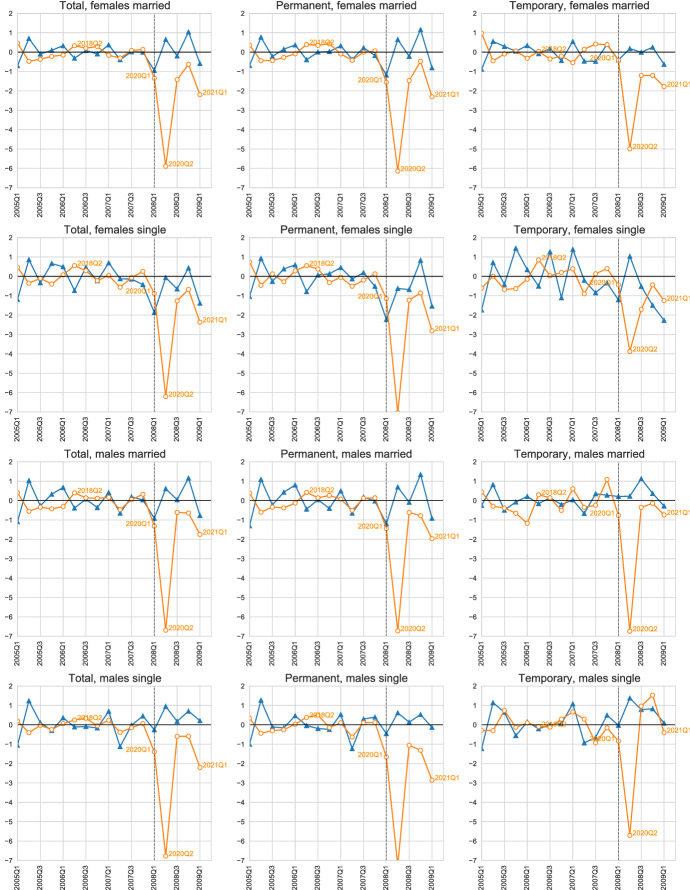
*Absolute* response of *hours per worker* by gender and marital status in the Great Recession and COVID-19, prime-age adults. *Notes*: Aggregate hours of Married Females means the sum of all hours worked by married female. All variables are plotted as *absolute* deviations from a predicted trend that uses data up to the onset of recessions. Note that we have shifted the Covid-19 Recession in time in order to normalize the onset of the Covid-19 Recession to that of the Great Recession. Prime-age adults are de1ned between 30 and 50 years old

## Mathematical appendix

Although the mathematical problem is simple, because it is standard static optimization, for completeness we derive here the results.

### Proof of Result 1

This can be simple constrained optimization. Rephrasing the problem, slightly generalized, in Lagrangian formulation

$$\begin{aligned}&{\mathcal {L}}(H_P^+, H_P^-, H_T, \mu ^+, \mu ^-) \\&\quad = p K^{\alpha }\Big (\eta _P H_P^\frac{\sigma -1}{\sigma } + \eta _T H_T^\frac{\sigma -1}{\sigma }\Big )^\frac{(1-\alpha ) \sigma }{\sigma -1}- w_T H_T - w_P (H_P^0+H_P^+)\\&\qquad -w_P(1-\tau )H_P^ - +\mu ^+ H_P^+ - \mu ^- H_P^-, \end{aligned}$$

where $H_P=H_P^0+H_P^+-H_P^-$, and the last line refers to inequality constraints

$$\begin{aligned} \mu ^+H_P^+\ge 0, \text { with multiplier } \mu ^+\ge 0 \\ \mu ^-H_P^-\ge 0, \text { with multiplier } \mu ^-\ge 0. \end{aligned}$$

First-order conditions are (4) and

10 $$\begin{aligned} \frac{\mathrm{d}{\mathcal {L}}}{\mathrm{d}H_P^+}=&p \ \eta _P (1-\alpha )K^{\alpha } \bigg (\eta _P + \eta _T \Big (\frac{H_T}{H_P}\Big )^{\frac{\sigma -1}{\sigma }}\bigg )^{\frac{1-\sigma \alpha }{\sigma -1}} \cdot H_P^{-\alpha }-w_P + \mu ^+=0. \end{aligned}$$

11 $$\begin{aligned} \frac{\mathrm{d}{\mathcal {L}}}{\mathrm{d}H_P^-}=&p \ \eta _P (1-\alpha )K^{\alpha } \bigg (\eta _P + \eta _T \Big (\frac{H_T}{H_P}\Big )^{\frac{\sigma -1}{\sigma }}\bigg )^{\frac{1-\sigma \alpha }{\sigma -1}} \cdot H_P^{-\alpha }-w_P(1-\tau ) + \mu ^-=0. \end{aligned}$$

It follows directly that it cannot be that $\mu ^{+}=\mu ^{-}=0$, and hence, we cannot have $H_P^{+}>0, H_P^{-} > 0$ simultaneously, and hence, this possibility of the more general formulation will never be optimal. In addition, it follows from the above two equations and (4) that

12 $$\begin{aligned} \frac{\eta _T}{\eta _P}\left( \frac{H_T}{H_P}\right) ^{-1/\sigma }&\le \frac{w_T}{w_P(1-\tau )}, \end{aligned}$$

13 $$\begin{aligned} \frac{\eta _T}{\eta _P}\left( \frac{H_T}{H_P}\right) ^{-1/\sigma }&\ge \ \frac{w_T}{w_P}, \end{aligned}$$

where (given complementary slackness for $\mu ^+, \mu ^-$)

$$\begin{aligned} \frac{w_T}{w_P}< \frac{\eta _T}{\eta _P}\left( \frac{H_T}{H_P}\right) ^{-1/\sigma }< \frac{w_T}{w_P(1-\tau )} \Rightarrow H_P=H_P^0. \end{aligned}$$

Using the properties of the production function, in particular the concavity of the production function and the constant returns to scale of the hours aggregator, the ratio $\frac{\eta _T}{\eta _P}\left( \frac{H_T}{H_P^0}\right) ^{-1/\sigma }$ is decreasing in $H_T$. Hence, we can split the domain of $H_T$ into three parts, with boundaries $\underline{H_T}, \overline{H_T}$, with $\underline{H_T}$ implicitly defined by (12) holding with equality at $H_P^0$, and $\overline{H_T}$ similarly for (13). Now consider the first-order condition (4) for $H_T$, reformulated with $w_T/p$ on the LHS. The RHS incorporates the three segments for $H_T$, and is a decreasing function, for a given $H_0$. Hence, there is a level ${\underline{p}}$ where this FOC holds at $\underline{H_T}$, this is ${\underline{p}}$, similarly for ${\overline{p}}$. For any $p<{\underline{p}}$, (12) holds with equality, and $H_P$ adjusts as well. (This equation coincides with Eq. (6, analogously for $p>{\overline{p}}$ for Eq. (8).) $\square$

## Data: unadjusted data time series (Fig. 2)

Figure 12 shows the raw series behind Fig. 2.

## Data: detailed prime-age patterns across marital status and gender

In this section, we report the aggregate hours response by gender and contract type for prime-age workers, in Fig. 13. The patterns look very similar by gender. Digging deeper, we split this marital status in Fig. 14. We see here that responses across married men in permanent contracts appear similar to married men, but this obscured that there are proportionally less married women working; per working woman in a permanent contract, the loss of hours is higher. Going to employment in Fig. 15, we observe the strong reaction for married women in permanent contracts in the COVID-19 Recession, where there was none in the Great Recession. This reaction was immediate, while married men in permanent had employment that reacted too, but build over multiple quarters. Interestingly, married women in temporary contracts reacted less than married men in the same contract, in terms of employment. Finally, in Fig. 16 we plot the hours per worker response. The first-order observation is large hours per worker responses across permanent and temporary, for all categories (with the possible exception of women on temporary contracts, which show a large response that is nonetheless somewhat more dampened compared to the others).

## Footnotes

1 Note that firms can decide not to renew temporary contracts. This choice does not show as a layoff, but it increases the flow out of employment.

2 Indeed, the fluctuations in aggregate hours—the product between employment and hours per worker—are understood to be largely driven by employment (Cooley and Prescott 1995).

3 Hospitality and retail are also sectors with a naturally higher share of temps, so we could expect employment loses on impact as large as construction in the Great Recession. Or even higher, as forced closures could translate into business shutdowns, which would affect perms much more than in the Great Recession.

4 Short-time work policies are defined by the 2020 European Commission as *“public programs that allow firms experiencing economic difficulties to temporarily reduce the hours worked while providing their employees with income support from the State for the hours not worked.”*

5 Isolating the effects of an specific COVID-19 policy is challenging given the quarterly frequency of our data and the fact that these policies (e.g., national lockdown and ERTEs) were instated within the same quarter.

6 Although limited by its static nature, our discussion points to the importance of using a dynamic setting to assess both the role of firing costs and the effects of COVID-19 policy as a path for future research. Indeed, the available evidence strongly suggests the use of explicitly dynamic, stochastic, frictional models of dual labor markets, in the line of, e.g., Bentolila et al. (2012a) or Costain et al. (2010), which if adapted to the current policy environment could help us understand better the situation and policy choices in COVID times.

7 As explained later, if firms lay off employees subject to ERTEs, they must return the subsidies received in full.

8 The impact is very different across industries, as ERTEs alter the challenges faced by parents: Those in ERTEs can tend to children at home without losing their jobs. Those who cannot take leave face harsher constraints. We are currently working on this issue in a separate project.

9 This is similar to other rotating labor force panels with some differences in the frequency and the length of the panel. For example, in the Current Population Survey in the USA individuals are interviewed four consecutive months in year *t* and, after a span of 8 months without interviews, individuals are interviewed for an additional set of four consecutive months in year $t+1$.

10 Given that hours per worker is a margin that can be used to adjust aggregate hours, we focus on the (self-)reported actual hours worked (last week). That is, “Number of effective hours devoted to this [main] job last week” or *Numero de horas efectivas que dedico a este trabajo la semana pasada* in Spanish.

11 See Conde-Ruiz et al. (2010) for more details on the impact of this reform.

12 A German tourist visiting the Canary Islands.

13 According to the Spanish Economic Association, the first recession lasted from 2008Q2 to 2009Q4 and the second from 2010Q4 to 2013Q2.

14 The cost of this measure is still unclear after a year into the crisis. Part of the reason for this is that the government is not spending anything directly as a result of the ERTE, but rather the workers are consuming their allowance. Therefore, estimates of the cost vary considerably even by government estimates. A generally accepted estimate is 4,000 million euros on average per month until September 2020 and about 3,500 million euros per month thereafter. The number falls down as workers under ERTE fall. Just by this estimate, it can be seen that the money invested in ERTEs is far superior to other credit or subsidies firms received in this period.

15 There are special provisions for workers with more than one active job and workers affected between October 2020 and January 2021. For the duration of the COVID short-time work, firms with less than 50 employees do not pay social contributions for the worker, and large firms pay only 25% of the regular amount. For more details, see Real Decreto-ley 8/2020 of March 17 (BOE 73)

16 Contract expiration takes into account the time added because of the duration of an ERTE. The right to reduced hours for workers with family care needs is also reinforced.

17 See Real Decreto-ley 7/2020 of March 12 (BOE 65).

18 The labor Force Survey (EPA) in Spain is conducted in such a way that we cannot identify the week that interviews take place within a quarter.

19 Recently, Aleman et al. (2020) estimate the effectiveness of the national lockdown in Spain and argue that the lockdown came with a substantial delay in terms of lives saved. The delayed government response was not unique to Spain. Most European countries had a delayed response to the crisis, most notably the UK.

20 The full list of essential industries can be found in the text of the *Real Decreto-ley 10/2020*, from 29 March, which can be found here: https://www.boe.es/boe/dias/2020/03/29/pdfs/BOE-A-2020-4166.pdf.

21 The working-age population does not adjust for those who are out of the labor force due to education, sickness, early retirement, etc.

22 This information is taken from a variable in the EPA that records the *“Reason for not having worked in the reference week, if employed”*. One of the answers is being subject to ERTE, and that is what we count as furloughed workers: those classified as employed (either perms or temps) that reported no hours and cited as the reason an ERTE. There is also a partial ERTE question (so workers do work but less hours than usual) but we do not use these as they are not the ones that the government introduced as a response to the COVID-19 emergency.

23 As per our discussion in Sect. 3.2, the response of aggregate hours summarizes the joint response of employment and hours per worker.

24 Note that adding capital as complement to the labor composite in brackets in (2) does not change our analysis since we are going to be interested in the parameter $\sigma$ that is pinned down by the relative wages and relative labor inputs of permanent workers and temps.

25 Note that while firms face a static trade-off between the two types of labor here, this can be thought to capture (though in very reduced form) some of the firms’ *dynamic* portfolio choice regarding a stable “permanent” workforce (which, e.g., can acquire skills with firm tenure to perform tasks better) and a “temporary” workforce (which provides flexibility in response to shocks), with parameter $\sigma$ capturing how easily this portfolio can be varied.

26 Thus, implicitly in modeling the Great Recession, we assume that hours reduction comes mainly in the form of employment (i.e., number of workers) reduction, costly in case of permanent workers and not so in case of temporary workers. In discussing COVID times subsequently, we consider the same dimension, hours, but now it can adjusted without necessary dismissals and without differential costs for the permanents. For temporary workers, hours adjustment can be either in the form of an ERTE-hours adjustment per worker or a dismissal—in reality both margins have been used (as also visible in Sect. 3).

27 Though, of course, it must be said that the pattern is richer in the data, where retracing involves not only changes in $H_P$ but also the changing $H_T/H_P$ ratio.

28 The observation that, in the data, the recovery in the third quarter of 2020 is associated with a flatter slope from 2020Q2 to 2020Q3 in Fig. 5 than from 2020Q1 to 2020Q2 is not necessarily contradictory with this mechanism per se: In the data adjustment did not exclusively happen through ERTE, but to a lesser extent through employment reductions, with the former force also of some relevance in 2020Q2, as Fig. 3 shows. With the adjustment of ERTEs changing hours in 2020Q3 more dominant than employment changing in 2020Q3, the subsequent adjustment of $H_P$ with less effect on $H_T/H_P$ is then also more in line with the model, i.e., recovery with a flatter line in $(H_P, H_T/H_P)$-space.

29 The model could otherwise be generalized to smooth out the angular -shape in Fig. 6. Intuitively, heterogeneous firing cost across workers, or a probability that some permanent workers would have to be dismissed at the end of the period in any case (lowering the opportunity cost of immediate dismissal) could help with this.

30 Some of this ‘dynamic’ imperfect substitutability of both types of labor could be thought as captured reduced form by the complementarity parameter in the production function. Modeling this explicitly, however, could widen the scope of the model, to address question regarding ERTEs/short-time work and labor misallocation. We thank a referee for pointing this out.

31 Hours are once again normalized by the entire working-age population. As before, one hour on this graph corresponds to one hour per week of the representative working-age person.

32 As a result, it is hard to tell from this figure if there could have been an effect of the 2012 labor market reform on the propensity for permanent dismissal in COVID times.

33 Fully dynamic stochastic models can capture the trade-offs between firing flexibility, the benefits of employment stability, and productive misallocation, by giving an explicit role to time and the environment that changes with it (cf. the papers mentioned in Sect. 2.2).

34 This leaves open the possibility that there is a link between structural transformation and these adjustment dynamics: Sectors that are in longer-term decline adjust perm hours earlier in the recession.

35 We thank the referees for pointing this channel to us.

36 The role of ERTEs here is significant: If parents are in ERTEs, they can easily stay home and take care of the children. Parents who need to work—particularly those who work outside of the house—are the ones that are going to have binding labor supply responses. We are exploring this potential effect of ERTEs in another paper.

37 See “Appendix” for the analogue of Fig. 11 in absolute deviations. Relative changes make more sense in this setting, since there are more married people in the sample.

38 The EPA does not provide information on children present in the household, but it does provide information on marital status on age—we therefore focus on married individuals between 30 and 50 years of age.

39 See “Appendix C” for the pictures on hours per worker and employment.
